# Supplementary material for: Tumor cell E-selectin ligands determine partialefficacy of bortezomib on spontaneous lung metastasis formation of solid human tumors in vivo
Source: Mol Ther. 2022 Jan 12;30(4):1536–52. doi: 10.1016/j.ymthe.2022.01.017 (PMC9077315; doi:10.1016/j.ymthe.2022.01.017)
Supplement: Document S1. Figures S1–S4, Table S1, and Supplemental methods [file mmc1.pdf]

YMTHE, Volume *30*

Supplemental Information

Tumor cell E-selectin ligands determine partial efficacy of bortezomib on spontaneous lung metastasis formation of solid human tumors *in vivo*

Tobias Lange, Ursula Valentiner, Daniel Wicklein, Hanna Maar, Vera Labitzky, Ann-Kristin Ahlers, Sarah Starzonek, Sandra Genduso, Lisa Staffeldt, Carolin Pahlow, Anna-Maria Dück, Christine Stürken, Anke Baranowsky, Alexander T. Bauer, Etmar Bulk, Albrecht Schwab, Kristoffer Riecken, Christian Börnchen, Rainer Kiefmann, Valsamma Abraham, Horace M. DeLisser, Timo Gemoll, Jens K. Habermann, Andreas Block, Klaus Pantel, and Udo Schumacher

Supplemental Figures and Legends

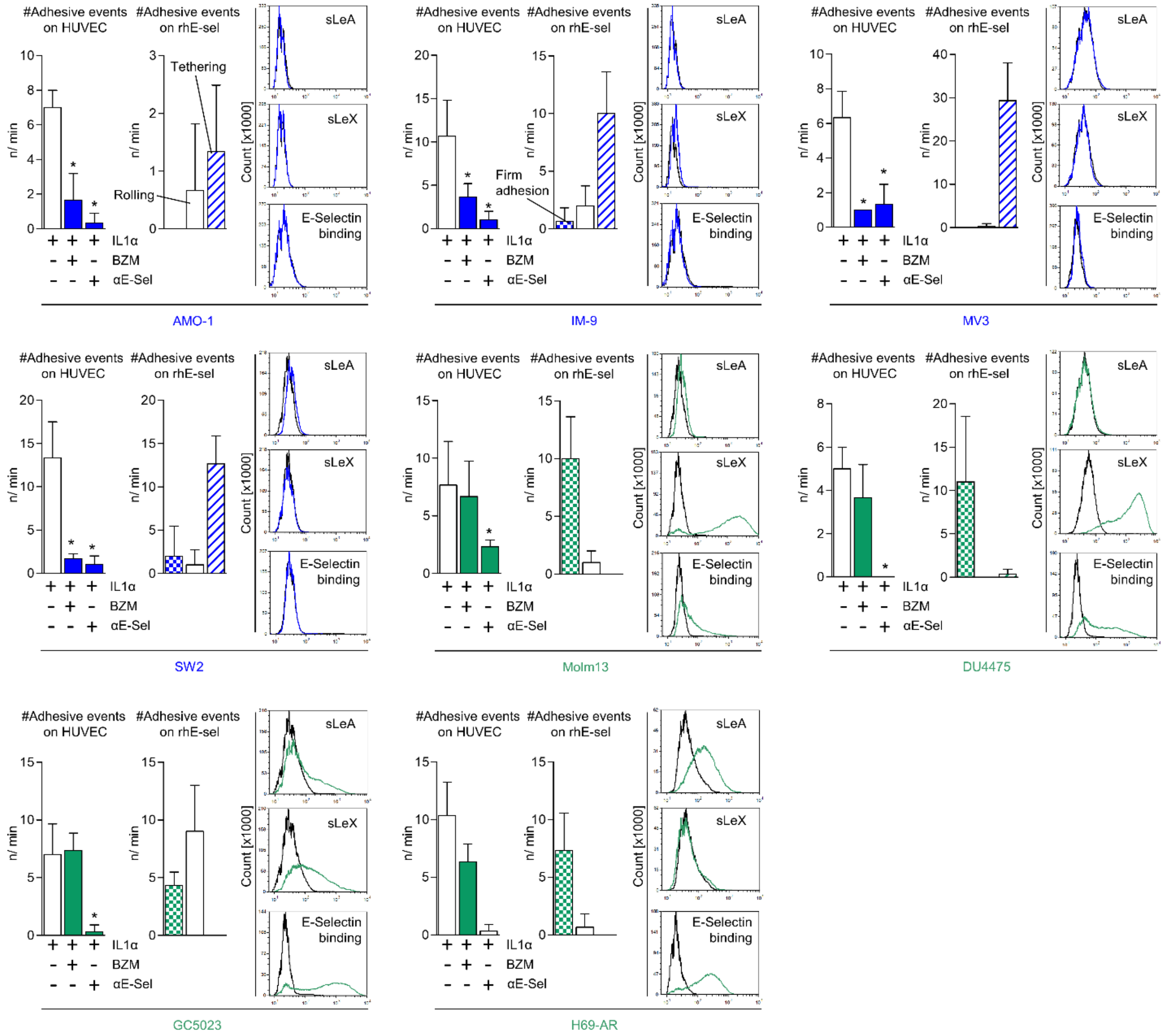

Figure S1: Verification of the relationship between adhesive properties of the tumor cells and anti-adhesive efficacy of BZM by further human tumor cell lines. Numbers of adhesive events of indicated tumor cells under flow conditions on ECs treated +/-IL-1α +/-BZM +/-E-selectin-blocking antibody as well as on immobilized rhE-selectin. Representative histograms of tumor cell surface sLeA/X expression and static E-selectin binding capacity. Bar charts represent mean ± SD of three replicates; black lines in histograms represent isotype controls (sLeA, sLeX) or binding of IgG1-Fc (rhE-selectin binding); *p<0.05.

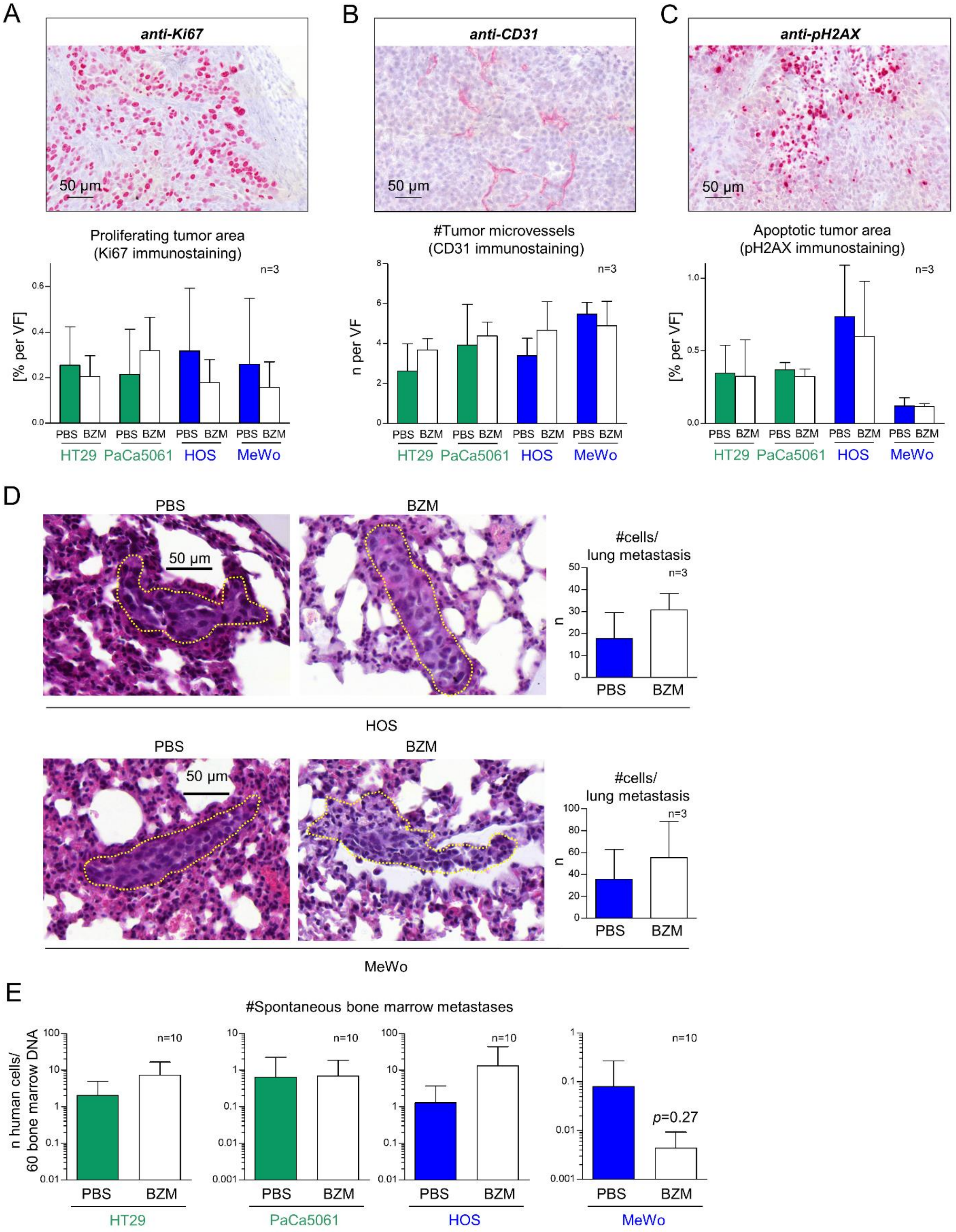
A
anti-Ki67
50 µm
Proliferating tumor area
(Ki67 immunostaining)
n=3
[% per VF]
PBS BZM PBS BZM PBS BZM PBS BZM
HT29 PaCa5061 HOS MeWo
B
anti-CD31
50 µm
#Tumor microvessels
(CD31 immunostaining)
n=3
n per VF
PBS BZM PBS BZM PBS BZM PBS BZM
HT29 PaCa5061 HOS MeWo
C
anti-pH2AX
50 µm
Apoptotic tumor area
(pH2AX immunostaining)
n=3
[% per VF]
PBS BZM PBS BZM PBS BZM PBS BZM
HT29 PaCa5061 HOS MeWo
D
PBS
BZM
50 µm
#cells/
lung metastasis
n=3
n
PBS BZM
HOS
PBS
BZM
50 µm
#cells/
lung metastasis
n=3
n
PBS BZM
MeWo
E
#Spontaneous bone marrow metastases
n human cells/
60 bone marrow DNA
n=10
p=0.27
PBS BZM
HT29
PaCa5061
HOS
MeWo

Figure S2: Exclusion of potential 'off-target' effects of BZM. Immunostaining-based quantification of Ki67-positive xenograft primary tumor cells (A), CD31-positive xenograft primary tumor microvessels (B) and p2H2AX-positive xenograft primar tumor cells in the indicated xenograft models and treatment groups (C). Number of cells per lung metastasis in PBS vs. BZM-treated mice carrying the annotated s.c. xenografts (D). Number of spontaneous bone marrow metastases in PBS vs. BZM-treated mice bearing the indicated s.c. xenografts (E). Bone marrow endothelium is known to constitutively express E-selectin [17]. Bar charts in (A-C) represent mean ± SD from n=3, each containing up to 27 viewing fields per tumor. Bar charts in (D) represent mean ± SD of n=3. Bar charts in (E) represent mean ± SD of n=10.

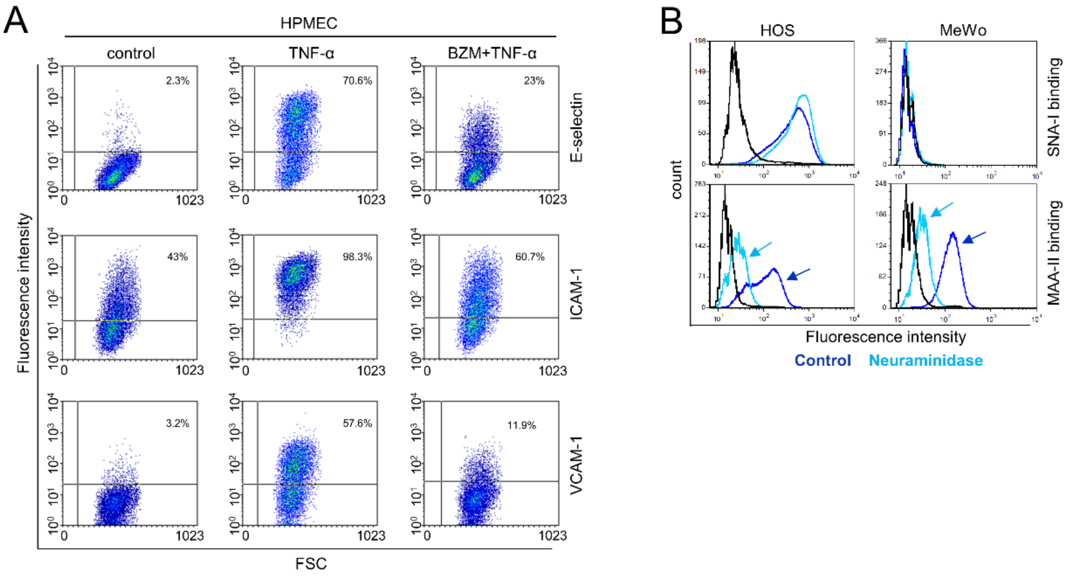

Figure S3: BZM effects on cytokine-mediated induction of CAMs in HPMEC and detection of α-2,3- and α-2,6-sialic acid on sLeA/X-negative tumor cells. Flow cytometric analyses of E-selectin, ICAM-1 and VCAM-1 after TNF-α stimulation and pre-incubation with BZM in HPMEC (A). MAA-II and SNA-I lectin binding towards HOS and MeWo cells before (dark blue) and after (light blue) neuraminidase treatment as indicated (B).

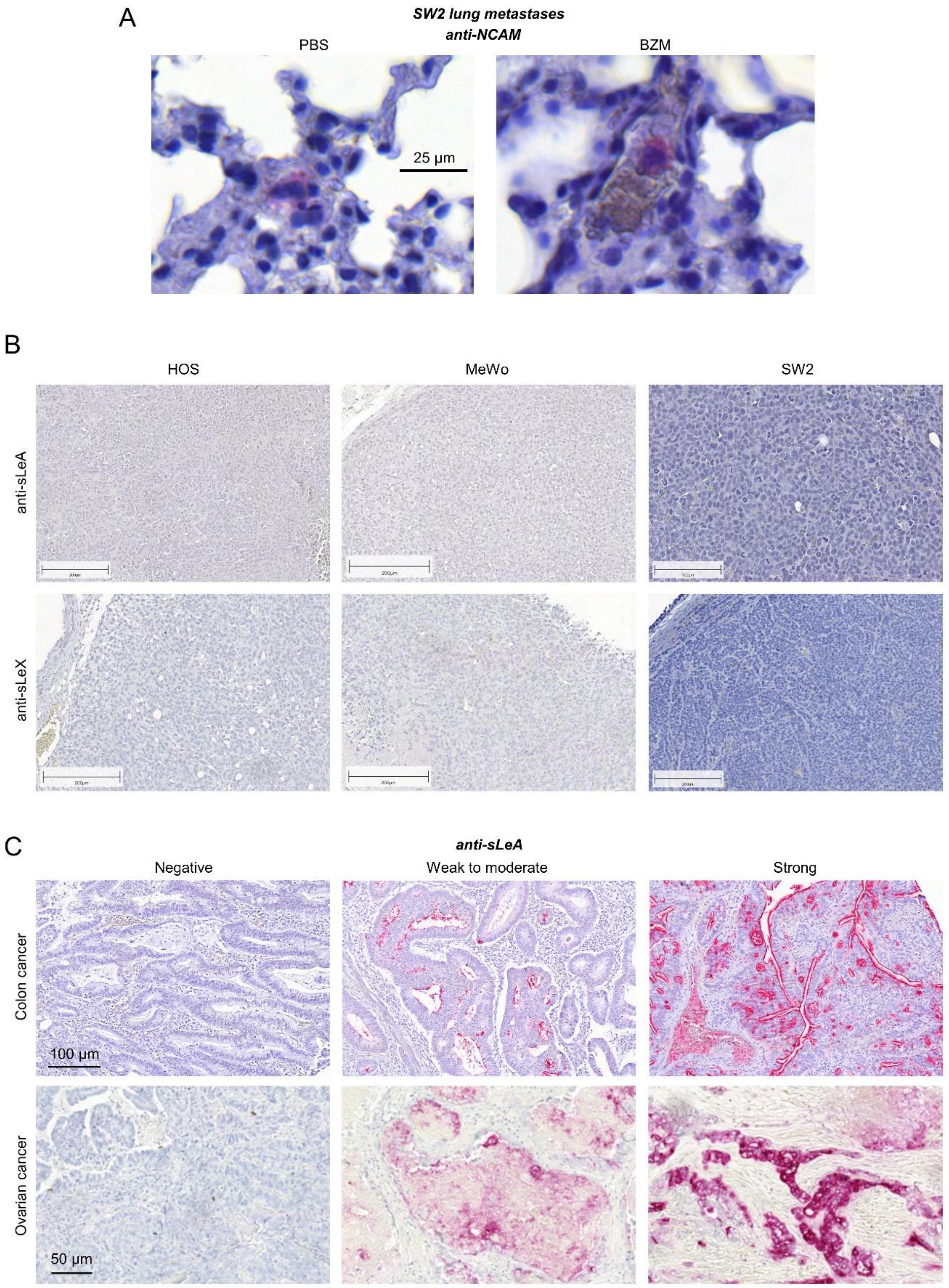

Figure S4: Detection of SW2 single cell metastases and determination of sLeA. Detection of human small cell lung cancer SW2 cells in the lungs of mice bearing s.c. xenograft tumors. In both

groups (PBS vs. BZM), only single cells could be found in the lungs (visualized by anti-NCAM immunostaining) (A). Other than SKOV3 (Fig. 6D), HOS, MeWo and SW2 xenografts fail to upregulate sLeA or sLeX *in vivo* (B). Detection of differential sLeA levels in primary patient material of colon and ovarian cancer by IHC (B).

Table S1: qPCR profiler array human glycosylation/ glucosidase genes, see Fig. 4E.

	2^-ΔCt			Fold Regulation	
Gene symbol	HT29	HOS	MeWo	HOS *vs.* HT29	MeWo *vs.* HT29
A4GNT	2.2E-04	5.4E-05	5.8E-04	-4.04	2.65
AGA	1.5E-02	3.7E-03	2.0E-02	-3.95	1.34
B3GALTL	2.5E-02	9.1E-03	2.8E-02	-2.68	1.13
B3GNT2	8.1E-02	2.5E-02	7.5E-02	-3.28	-1.08
B3GNT3	4.3E-02	1.7E-03	2.4E-03	-24.84	-17.9
B3GNT4	7.1E-04	6.3E-04	1.1E-03	-1.11	1.55
B3GNT8	5.7E-04	2.7E-04	1.5E-03	-2.09	2.58
B4GALT1	1.6E-01	3.2E-02	1.0E-01	-5.08	-1.6
B4GALT2	6.5E-02	4.2E-02	1.1E-01	-1.56	1.62
B4GALT3	5.8E-02	2.6E-02	6.1E-02	-2.19	1.05
B4GALT5	7.8E-02	5.3E-02	1.5E-01	-1.47	1.91
C1GALT1	1.2E-01	4.5E-02	9.7E-02	-2.66	-1.24
C1GALT1C1	5.2E-02	4.1E-02	4.9E-02	-1.27	-1.05
EDEM1	2.3E-02	5.7E-02	1.1E-01	2.45	4.91
EDEM2	2.5E-02	1.9E-02	3.4E-02	-1.29	1.39
EDEM3	4.7E-02	3.4E-02	3.4E-02	-1.38	-1.38
FUCA1	5.3E-02	3.9E-03	1.6E-02	-13.68	-3.27
FUCA2	9.8E-02	9.5E-02	2.9E-01	-1.02	2.98
FUT11	2.4E-02	2.1E-02	7.9E-02	-1.12	3.26
FUT8	4.5E-03	1.6E-01	3.9E-02	34.89	8.67
GALNT1	2.3E-02	8.2E-02	1.6E-01	3.56	6.9
GALNT10	7.8E-03	4.8E-02	7.9E-03	6.12	1.01
GALNT11	1.5E-02	5.9E-03	5.4E-02	-2.59	3.52
GALNT12	1.1E-01	2.6E-03	7.7E-03	-41.49	-14.24
GALNT13	5.7E-05	2.9E-05	4.1E-05	-1.99	-1.38
GALNT14	5.5E-02	1.6E-02	3.7E-05	-3.42	-1491.45
GALNT2	2.4E-02	3.7E-02	2.2E-01	1.51	9.11
GALNT3	1.8E-01	2.1E-05	1.4E-01	-8569.52	-1.26
GALNT4	1.5E-02	6.0E-03	6.9E-03	-2.52	-2.19
GALNT6	1.4E-03	1.1E-02	4.0E-04	7.33	-3.56
GALNT7	4.6E-02	3.3E-02	2.2E-02	-1.38	-2.05
GALNT8	2.0E-04	1.6E-05	3.7E-05	-12.25	-5.43
GALNT9	1.8E-04	1.4E-04	5.6E-04	-1.31	3.15
GALNTL1	1.1E-04	3.7E-03	1.3E-04	34.41	1.15
GALNTL5	4.9E-05	1.9E-05	8.3E-05	-2.54	1.7
GALNTL6	2.1E-05	1.6E-05	6.9E-05	-1.3	3.22
GANAB	5.0E-01	4.3E-01	6.5E-01	-1.16	1.29
GCNT1	1.2E-03	1.5E-03	3.4E-03	1.21	2.72
C2GNT2	9.2E-01	1.7E-04	1.1E-03	-5499.17	-856.61
GCNT4	2.9E-04	6.3E-04	3.9E-04	2.16	1.32
GLB1	6.8E-02	5.2E-02	5.5E-02	-1.3	-1.24

GNPTAB	2.3E-02	1.2E-02	2.4E-01	-1.87	10.46
GNPTG	3.3E-02	1.7E-02	8.8E-02	-1.91	2.68
HEXA	7.7E-03	1.1E-02	1.3E-01	1.46	16.53
HEXB	1.3E-01	6.5E-02	3.1E-01	-1.91	2.47
MAN1A1	5.9E-02	4.1E-02	6.8E-04	-1.45	-86.97
MAN1A2	3.3E-02	1.1E-02	4.6E-02	-3.06	1.42
MAN1B1	6.2E-02	2.9E-02	8.4E-02	-2.15	1.35
MAN1C1	1.6E-04	2.3E-03	7.4E-04	14.57	4.68
MAN2A1	4.2E-02	1.9E-02	3.6E-02	-2.19	-1.16
MAN2A2	2.4E-02	4.3E-03	4.6E-02	-5.59	1.91
MAN2B1	1.6E-02	2.1E-02	1.7E-02	1.3	1.09
MANBA	1.9E-02	5.0E-03	2.3E-02	-3.71	1.24
MGAT1	2.8E-02	1.5E-02	7.9E-02	-1.83	2.86
MGAT2	6.8E-03	2.2E-02	1.6E-02	3.17	2.34
MGAT3	3.7E-03	1.2E-04	3.7E-05	-29.54	-98.53
MGAT4A	3.6E-03	1.5E-04	1.3E-02	-23.34	3.54
MGAT4B	1.8E-01	5.7E-02	1.1E-01	-3.08	-1.66
MGAT4C	2.1E-05	1.6E-05	3.7E-05	-1.3	1.72
MGAT5	9.0E-02	2.2E-02	7.4E-02	-4.09	-1.21
MGAT5B	1.6E-04	1.0E-02	1.0E-01	63.77	638.03
MOGS	4.6E-02	3.0E-02	6.3E-02	-1.54	1.36
NAGPA	1.4E-03	1.3E-03	3.2E-03	-1.08	2.21
NEU1	9.2E-02	4.1E-02	1.7E-01	-2.27	1.83
NEU2	4.1E-04	1.2E-04	7.9E-04	-3.37	1.94
NEU3	9.8E-03	6.6E-03	6.8E-02	-1.47	6.95
NEU4	2.5E-04	8.9E-05	2.4E-04	-2.83	-1.03
OGT	7.9E-02	4.7E-02	4.9E-02	-1.67	-1.6
POFUT1	3.6E-02	3.0E-02	2.0E-02	-1.19	-1.8
POFUT2	2.6E-02	1.8E-02	1.0E-02	-1.42	-2.64
POMGNT1	5.0E-02	3.9E-02	7.8E-02	-1.27	1.56
POMT1	8.2E-04	6.5E-04	2.7E-02	-1.25	32.84
POMT2	1.0E-02	2.2E-02	5.1E-02	2.16	5.16
PRKCSH	2.0E-01	1.8E-01	3.0E-01	-1.09	1.54
ST3GAL1	2.0E-01	9.9E-03	1.1E-02	-20.46	-17.78
ST3GAL2	4.7E-02	2.0E-02	4.3E-02	-2.35	-1.1
ST6GAL1	9.5E-03	1.9E-02	1.2E-03	2.02	-8.06
ST6GALNAC1	2.2E-03	1.6E-05	6.1E-05	-132.05	-35.81
ST8SIA2	2.2E-05	2.4E-05	8.8E-05	1.11	4.04
ST8SIA3	9.2E-05	1.2E-04	6.2E-05	1.27	-1.48
ST8SIA4	2.1E-05	6.2E-04	7.6E-03	28.94	353.97
ST8SIA6	2.1E-05	3.2E-05	2.3E-03	1.48	105.96
UGCGL1	5.3E-02	6.2E-02	1.2E-01	1.16	2.32
UGCGL2	3.1E-02	1.1E-02	2.6E-02	-2.79	-1.17

Supplemental Methods

Human tumor cell lines

HT29 human colorectal cancer cells and DU4475 human breast cancer cells were purchased from ECACC (Porton Down, UK). HOS osteosarcoma[1] as well as MeWo and MV3 melanoma[2] cells were kindly provided by the Dept. of Pediatric Hematology and Oncology (University Medical Center Hamburg-Eppendorf, UKE) and the Dept. of Dermatology at UKE, respectively. The human pancreatic cancer cell line PaCa5061[3] and gastric cancer cell line GC5023[4] were established in the Dept. of General, Visceral and Thoracic Surgery at UKE. The human small cell lung cancer cell lines SW2 and H69-AR were kind gifts from Prof. Zangemeister-Wittke (Institute of Pharmacology, University of Bern, Switzerland). The human multiple myeloma cell lines AMO-1 and IM-9 were kindly provided by Prof. M. Binder (Dept. of Oncology and Hematology, UKE). The human acute myeloid leukemia cell line Molm13 was provided by Dr. J. Wellbrock (Depot. of Oncology and Hematology, UKE). Human SKOV3 ovarian cancer cells were obtained from ATCC (Manassas, USA) and cultured in McCoy's 5A + L-glutamine supplemented with 10% FCS and 1% penicillin/ streptomycin (Gibco). All other human tumor cell lines were cultured in RPMI-1640 + L-glutamine (Gibco), supplemented with 10% FCS and 1% penicillin/ streptomycin and were kept under standard conditions (37°C, 95% H_2O-saturated atmosphere, 5% CO_2).

Human primary tumor models

The primary human colon cancer model PT457 was derived from a primary adenocarcinoma of the colon which was surgically resected from a male Caucasian in 2014. The PT1003 model was

established from a surgically resected, chemo-naïve liver metastasis of a colon sigmoideum adenocarcinoma from a 73 years-old male Caucasian. For spheroid culture, the protocol published by Jeppesen et al. was used.[5] Slight modifications of this protocol were the use of a 100 µm mesh size cell strainer for preparation and the use of Matrigel-coated 24-well plates for subsequent cultivation. Tumoroids were growing in 30-50µm domes surrounded by 1 mL stem cell medium. The use of patient material for research purposes was approved by the local ethics committee (Ärztekammer Hamburg, project PV4753, Sept 2nd, 2014).

Primary endothelial cells

Human umbilical vein endothelial cells (HUVEC) and human pulmonary microvascular endothelial cells (HPMEC) were obtained from PromoCell and cultured as described.[6,7] Murine pulmonary endothelial cells (MPEC) were freshly isolated as illustrated in Fig. 2D and previously described.[8] All experiments with primary cells were performed within the first six passages.

qRT-PCR for pulmonary Sele, Vcam1 and Icam1 expression

To analyze effects of the BZM treatment on pulmonary *Sele*, *Vcam1* and *Icam1* expression, xenograft tumor-bearing mice treated with PBS or BZM were sacrificed, lungs were resected and total RNA was extracted using a standard phenol/chloroform protocol and transcribed to cDNA using the cDNA RT² Easy First Strand Synthesis Kit (Qiagen). cDNA was used for qRT-PCR in a StepOnePLus system (Applied Biosystems, Thermo Fisher). *Gapdh* was used as housekeeping control. Nucleotide sequences of SYBR® Green primers were as follows:

Sele-fwd GGCTTTAGCTTGCATGGCTC, *Sele*-rev CATCTTTCCCGGGACGTCAA; *Icam1*-fwd CCATCCATCCCAGAGAAGCC, *Icam1*-rev CACTGAGTCTCCAAGCCCAG; *Vcam1-f*wd GTCACGGTCAAGTGTTTGGC, *Vcam1*-rev TGTTCATGAGCTGGTCACCC.

Ex vivo lung perfusion model

SCID mice were left untreated (control) or treated with 1 μg rmTNFα i.p. (Peprotech) for 4h. TNFα-treated mice were pretreated with PBS (solvent control) or 1.25 mg/kg BZM 24h and 1h before TNFα injection. 4h after TNFα injection, mice were narcotized and the cardiorespiratory system was resected and extracorporeally perfused as described[9] and illustrated in Fig. 2E. In short, 12 weeks old mice were narcotized and a cannula was placed into the trachea, by which the lungs were held under static inflation with a gas mixture of 30 % O_2, 5% CO_2 and 65 % N_2. Hence, normoxic conditions were maintained during the experiment, but ventilatory lung excursions were concomitantly avoided. Afterwards, the Vv. cavae were ligated, cannulae were inserted into the pulmonary artery and the left atrium, and the cardiorespiratory system was resected *en bloc*. The lung vasculature was then perfused through the cannulae with a HEPES-buffered perfusate at 37°C. Under these conditions, the lung periphery was examined using real-time epifluorescent video microscopy (Zeiss Axiotech with a Zeiss 40x water emission objective [NA: 0.8], Photometrics CoolSnap HQ camera). Physiological blood flow conditions in the lung microvasculature were controlled by adding isolated red blood cells (RBC) to the perfusate. Afterwards, $1x10^6$ HOS cells (labeled with Calcein RedOrange) were added to the perfusate and their adhesive behavior was analyzed in each compartment of the pulmonary microcirculation, namely precapillary arterioles, capillaries and post-capillary venules. RBC velocity and HOS

tumor cell adhesion or arrest was analyzed from the digital recordings using image analysis software (MetaMorph, Molecular Devices).

Single cell force spectroscopy

To measure the adhesion force [nN] between cancer and endothelial cells, we applied single cell force spectroscopy (SCFS). As described earlier,[10-12] this method is based on atomic force microscopy (AFM) and allows to quantify the adhesion force (nN) between single cancer cells and endothelial cells. The CellHesion® software (JPK, SPM, version 4) directed the experiment. Analysis of the data was performed using JPK Data Processing (software version 4.3.18). Briefly, 1.5 x 10^5 human umbilical vein endothelial cells (HUVECs) were seeded overnight in a well of an ibidi Culture-Insert (Culture-Insert 2 well, #80209, ibidi, Germany). The next day, the Culture-Inserts were removed and the cells were supplemented for 4 h with fresh medium, with or without IL-1α (10 ng/ml). Tipless cantilevers (#ARROW-TL1x48-10, NanoWorld AG) were preincubated for 20 min in PBS containing 1 mg/ml wheat germ agglutinin (WGA; #L-9640, Sigma-Aldrich). Before starting the experiment, HUVECs were rinsed 3x with Ringer's solution (in mmol/l: NaCl 122.5, KCl 5.4, $CaCl_2$ 1.2, $MgCl_2$ 0.8, HEPES 10, and D-glucose 5.5, adjusted to pH 7.4 with 1 M NaOH) and maintained in this solution. Afterwards, 2 µl of a cancer cell suspension (~100 cells/µl) were added to a cell-free area within the dish containing the HUVECs. Next, the WGA-coated cantilever was guided under optical control over a single cancer cell and brought into contact with this cell for 6 s by pressing the cantilever onto the cell with a maximal loading force of 2 nN. To ensure that the cancer cells were attached to the cantilever and always had the same mechanical properties, we standardized this picking procedure.

For measuring the cell-cell adhesion forces, the parameters were changed to 2 s contact time with a maximum loading force of 1 nN. This time, the cantilever with the attached cancer cell was lowered under optical control onto a single HUVEC within the endothelial cell layer and after 2 s contact time the cantilever was lifted back to its starting position. The maximal adhesion force was determined from the resulting force-distance curve. Each cancer cell was brought into contact with 20 individual HUVECs (n=1). At least three independent experiments were performed for each condition (n≥3). Further parameters for the measurements were as follows: pulling length (z-length) was set to 100 µm (to ensure a complete separation of cells), velocity during approach and retraction was set to 5 µm/s. The spring constant was 0.03 N/m and had been corrected by the Standardized Nanomechanical AFM Procedure (SNAP).[13]

shRNA-mediated depletion of CD44, E-selectin, C2GNT2 and MGAT5

To test the functional importance of CD44 and MGAT5 (HOS and MeWo cells), E-selectin (HUVECs) and C2GNT2 (HT29 and PaCa5061 cells) the mRNAs of interest were stably depleted by lentiviral transduction of corresponding shRNAs. In case of the CD44 knockdown, a pLVX-puro vector was used and continuous selection with puromycin, FACS and limiting dilution cloning were performed. Finally, at least 5 clones showing the strongest depletion were pooled to avoid clonal effects. In case of the E-selectin and C2GNT2 knockdowns, cells were transduced with a pLVX-puro vector containing the shRNA of interest and subsequently selected with puromycin, but without FACS or limiting dilution cloning. In case of MGAT5, knockdown cells were transduced and selected in the same way, but additionally FACS-sorted based on cell surface PHA-L binding. In parallel, the ‘shControl’ cell lines were generated by transducing parental cells with a pLVX-puro vector containing a nonsense sequence (without mRNA target in mammalian

cells). The nucleotide sequences of the used shRNAs were: shCD44: 5’-GGCGCAGATCGATTTGAAT-3’; shE-selectin: 5’-CACACACCTGGTTGCAATT-3’; shC2GNT2 (GCNT3): 5’-CCGGGCTTAGAA-GAATACCTACGTTCTCGAGAACGTAGGTATTCTTCTAAGCTTTTTG-3’; shMGAT5: 5'-GATCCGGCGGAAATTCGTACAGATTTCAAGAGAATCTGTACGAATTTCCGCCTTTTT TACGCGTG-3'.

CAM and glycosyltransferase expression in human tumor cell lines

Commonly described E-selectin ligand carrier glycoproteins were tested on HT29, PaCa5061, HOS, and MeWo cells by flow cytometry using commercial antibodies against CD44 (B-F24), CD44v3 (3G5), CD24 (eBioSN3), CD43 (eBio84-3C1), ESL-1 (AE-6), MUC-1 (SantaCruz), PSGL-1 (FLEG), LGALS3BP (3G8). Isotype controls were used as appropriate. Tumor cells were marked dead or alive by propidium iodide staining immediately before flow cytometry.

RNA was extracted from HT29, HOS and MeWo cells and human glycosyltransferase qPCR profiler arrays performed as described.[14] In short, RNA concentrations were quantified using a NanoDrop spectrophotometer and 1 mg was processed to cDNA with RT2 First Strand Kit (SA Biosciences). Expression levels of 84 human glycosylation genes were determined with the Human Glycosylation RT^2 Profiler PCR Array (SA Biosciences) in a LightCycler 480 (Roche). Housekeeping genes for normalization and internal controls for genomic DNA contamination, RNA quality, and general PCR performance were included as well. Differences in C2GNT2 (GCNT3) expression as indicated by the qPCR profiler array were validated by WB as described.[15] Total protein extraction was made with RIPA lysis buffer. For protein detection, the following

antibodies were used: polyclonal rabbit anti-GCNT3 (C2GNT2), mouse monoclonal anti-HSC70 (B-6) (SantaCruz).

Lectin flow cytometry

α-2,3- and α-2,6-sialic acid residues within the human tumor cell glycocalyx were determined using biotinylated *Maackia amurensis* (MAA-II) and *Sambucus nigra* (SNA-I) lectin (Vector Labs.) at 10 µg/mL in lectin buffer (Tris-buffered saline +Ca^{2+}/ +Mg^{2+}) for 30 min at 4°C. As 'isotype' controls, lectins were applied after treating the tumor cells with periodic acid. Lectins were labeled with streptavidin-APC (Sigma) for flow cytometry. Tumor cells were treated with neuraminidase (see methods of main text) or left untreated prior to the lectin binding assay.

CD44 WB, enzymatic treatments and lectin pulldown assays

HOS and MeWo cell protein extracts from parental cell lines as well as MGAT5 control and knockdown derivatives were generated by scraping the cells in RIPA buffer (supplemented with protease inhibitor cocktail (Calbiochem) and x M vanadate, 30 min at 4°C, then 30 min centrifuged at 14,000 g at 4°C). Protein extracts were left untreated or treated with PNGase F (*Flavobacterium meningosepticum*, recombinant from *E. coli*; 1 U/ 10 µg protein overnight at 37°C) or sialidase (*Arthrobacter ureafaciens*, 1.25 mU/ 10 µg protein for 45 min at 37°C) before protein bands were separated using SDS-PAGE followed by anti-CD44 western blot analysis. Briefly, proteins were transferred to PVDF membranes and unspecific binding sites were blocked with blocking solution (StartingBlock™ Blocking Buffer) + 0.05% Tween 20 for 1h at RT. Primary antibody (R&D systems, clone 2C5, 1 µg/mL) was incubated for 1 h at RT. HRP-conjugated goat-anti-mouse was

used as secondary antibody and binding was detected using a chemiluminescence kit (Thermo Fisher). Additional tumor cell samples were treated with 2 µM synthetic swainsonine for 72 h prior to protein extraction (control: methanol). In addition, some protein extracts (+/- swainsonine or from MGAT5 control and knockdown derivatives) were incubated with DSL-agarose or PHA-L agarose (both from Vector Labs.) in a protein: lectin ratio of 10: 1 overnight at 4°C (rolling). After centrifugation and washing five times, the precipitated proteins were subjected to SDS-PAGE and CD44 WB as described above.

Immunohistochemistry

Immunohistochemistry for hNCAM was carried out on 4 µm lung tissue sections (FFPE) from SW2 xenograft mice. After de-paraffinization, antigen retrieval was performed with Dako S1699 Retrieval Solution at pH 6 in a microwave for 2 x 4 min. Final concentration of the primary antibody (Leica #NCL-CD56-1B6) was 0.88 µg/ml and visualization of bound antibody was performed with the Dako Real Detection System K5005.

5 µm sections of HT29, PaCa5061, HOS and MeWo xenograft tumors (FFPE) were stained with anti-Ki67 (Dako S1699 Retrieval Solution at pH 6, 85°C overnight; primary antibody: Dako #M7240, final concentration: 1.1 µg/ml; visualization with the Vectastain ABC-AP kit from Vector Labs.), anti-pH2AX (Dako S1699 Retrieval Solution at pH 6, 121°C, 10 min; primary antibody: abcam #ab81299, final concentration: 0.07 µg/ml; visualization with ABC-AP), and anti-CD31 (Dako S2367 Retrieval Solution at pH 9, 121°C, 10 min; primary antibody: abcam #ab28364, final concentration: 0.8 µg/ml, visualization with ABC-AP). For quantification of the Ki67 and pH2AX staining, the stained area was determined using Image J software (Color

Threshold Plugin) analyzing up to 27 viewing fields (200-fold magnification) depending on the entire size of the respective xenograft tumor section. Three samples per group (PBS vs. BZM) were considered per xenograft tumor entity (HT29, PaCa5061, HOS, MeWo). For quantification of the CD31 staining, the number of microvessels (CD31-positive) was counted in up to 20 viewing fields (200-fold magnification) per tumor depending on the tumor size and three samples were considered per group.

Immunohistochemistry for sLeA was performed on 5 µm sections of agar-embedded SKOV3 cells as well as SKOV3, HOS and MeWo xenograft tumors (FFPE). In addition, sLeA was stained on colon cancer and ovarian cancer surgical specimens. In short, sections were de-paraffinized and pre-treated with trypsin 0.1% in a water bath at 37°C for 10 min. The primary antibody was 121SLE from abcam (#ab3982) diluted 1:750 and bound antibody was visualized using the Dako Real Detection System K5005. Counterstaining of nuclei was performed with Mayer's hemalum solution for 3-5 s.

Patient material

Clinical colorectal cancer and ovarian cancer specimens were collected from surgically removed tissues adhering to guidelines of the local ethical review board and after written informed consent of the patients (ethics committee of the Universität zu Lübeck, approval #07-124). For sLeA immunohistochemistry, tissues of 60 colorectal cancers as well as 30 corresponding adjacent normal mucosa specimens from the same patients were selected. Tissue cores (diameter 1.5 mm) were implemented into a tissue microarray by using a semiautomated arrayer (TMArrayer, Pathology Devices, San Diego, USA) as described [16]. The ovarian cancer specimens were provided

by Professor Johannes Dietl (Department of Obstetrics and Gynecology, Würzburg University Hospital).

Statistics

Data are presented as means ± SD unless otherwise indicated. Student's t and Mann-Whitney-U tests were performed as appropriate to assess associations between variables. Statistical significance was assigned at 2-tailed *p*-values less than 0.05. Numbers of lung metastases were also evaluated by an analysis of covariance (ANCOVA) with the tumor weight (Fig. 6A) or growth period (Fig. 2B) as covariates. These statistical tests were carried out using IBM SPSS software (SPSS version 21.0 for Windows, IBM). All data were visualized using Graph Pad Prism 5.0 (Graph Pad Software, San Diego).

Supplemental References

1. McAllister, R.M., Gardner, M.B., Greene, A.E., Bradt, C., Nichols, W.W., and Landing, B.H. (1971). Cultivation in vitro of cells derived from a human osteosarcoma. Cancer *27*, 397-402.
2. Carey, T.E., Takahashi, T., Resnick, L.A., Oettgen, H.F., and Old, L.J. (1976). Cell surface antigens of human malignant melanoma: mixed hemadsorption assays for humoral immunity to cultured autologous melanoma cells. Proc Natl Acad Sci U S A *73*, 3278-3282.
3. Kalinina, T., Gungor, C., Thieltges, S., Moller-Krull, M., Penas, E.M., Wicklein, D., et al. (2010). Establishment and characterization of a new human pancreatic adenocarcinoma cell line with high metastatic potential to the lung. BMC Cancer *10*, 295. 1471-2407-10-295.
4. Starzonek, S., Maar, H., Labitzky, V., Wicklein, D., Rossdam, C., Buettner, F.F.R., et al. (2020). Systematic analysis of the human tumor cell binding to human vs. murine E- and P-selectin under static vs. dynamic conditions. Glycobiology. 10.1093/glycob/cwaa019.
5. Jeppesen, M., Hagel, G., Glenthoj, A., Vainer, B., Ibsen, P., Harling, H., et al. (2017). Short-term spheroid culture of primary colorectal cancer cells as an in vitro model for personalizing cancer medicine. PLoS One *12*, e0183074. 10.1371/journal.pone.0183074.
6. Richter, U., Schroder, C., Wicklein, D., Lange, T., Geleff, S., Dippel, V., et al. (2011). Adhesion of small cell lung cancer cells to E- and P-selectin under physiological flow conditions: implications for metastasis formation. Histochem Cell Biol *135*, 499-512. 10.1007/s00418-011-0804-4.
7. Lange, T., Kupfernagel, M., Wicklein, D., Gebauer, F., Maar, H., Brugge, K., et al. (2014). Aberrant presentation of HPA-reactive carbohydrates implies Selectin-independent metastasis formation in human prostate cancer. Clin Cancer Res *20*, 1791-1802. 10.1158/1078-0432.CCR-13-2308.
8. Fehrenbach, M.L., Cao, G., Williams, J.T., Finklestein, J.M., and Delisser, H.M. (2009). Isolation of murine lung endothelial cells. Am J Physiol Lung Cell Mol Physiol *296*, L1096-1103. 10.1152/ajplung.90613.2008.
9. Kiefmann, R., Rifkind, J.M., Nagababu, E., and Bhattacharya, J. (2008). Red blood cells induce hypoxic lung inflammation. Blood *111*, 5205-5214. 10.1182/blood-2007-09-113902.
10. Bulk, E., Kramko, N., Liashkovich, I., Glaser, F., Schillers, H., Schnittler, H.J., et al. (2017). KCa3.1 channel inhibition leads to an ICAM-1 dependent increase of cell-cell adhesion between A549 lung cancer and HMEC-1 endothelial cells. Oncotarget *8*, 112268-112282. 10.18632/oncotarget.22735.
11. Hofschroer, V., Koch, K.A., Ludwig, F.T., Friedl, P., Oberleithner, H., Stock, C., et al. (2017). Extracellular protonation modulates cell-cell interaction mechanics and tissue invasion in human melanoma cells. Sci Rep *7*, 42369. 10.1038/srep42369.
12. Lindemann, O., Rossaint, J., Najder, K., Schimmelpfennig, S., Hofschroer, V., Walte, M., et al. (2020). Intravascular adhesion and recruitment of neutrophils in response to CXCL1 depends on their TRPC6 channels. J Mol Med (Berl) *98*, 349-360. 10.1007/s00109-020-01872-4.
13. Schillers, H., Rianna, C., Schape, J., Luque, T., Doschke, H., Walte, M., et al. (2017). Standardized Nanomechanical Atomic Force Microscopy Procedure (SNAP) for Measuring Soft and Biological Samples. Sci Rep *7*, 5117. 10.1038/s41598-017-05383-0.
14. Lange, T., Ullrich, S., Muller, I., Nentwich, M.F., Stubke, K., Feldhaus, S., et al. (2012). Human prostate cancer in a clinically relevant xenograft mouse model: identification of beta(1,6)-branched oligosaccharides as a marker of tumor progression. Clin Cancer Res *18*, 1364-1373. 1078-0432.CCR-11-2900.
15. Oliveira-Ferrer, L., Kurschner, M., Labitzky, V., Wicklein, D., Muller, V., Luers, G., et al. (2015). Prognostic impact of transcription factor Fra-1 in ER-positive breast cancer: contribution to a metastatic phenotype through modulation of tumor cell adhesive properties. J Cancer Res Clin Oncol. 10.1007/s00432-015-1925-2.
16. Gemoll, T., Roblick, U.J., Szymczak, S., Braunschweig, T., Becker, S., Igl, B.W., et al. (2011). HDAC2 and TXNL1 distinguish aneuploid from diploid colorectal cancers. Cell Mol Life Sci *68*, 3261-3274. 10.1007/s00018-011-0628-3.
17. Schweitzer, K.M., Drager, A.M., van der Valk, P., Thijsen, S.F., Zevenbergen, A., Theijsmeijer, A.P., et al. (1996). Constitutive expression of E-selectin and vascular cell adhesion molecule-1 on endothelial cells of hematopoietic tissues. Am J Pathol *148*, 165-175.
