# Supplementary material for: Tumor cell E-selectin ligands determine partialefficacy of bortezomib on spontaneous lung metastasis formation of solid human tumors in vivo
Source: Mol Ther. 2022 Jan 12;30(4):1536–52. doi: 10.1016/j.ymthe.2022.01.017 (PMC9077315; doi:10.1016/j.ymthe.2022.01.017)
Supplement: Document S2. Article plus supplemental information [file mmc2.pdf]

Molecular Therapy
Original Article

Tumor cell E-selectin ligands determine partial efficacy of bortezomib on spontaneous lung metastasis formation of solid human tumors *in vivo*

Tobias Lange,[1,11] Ursula Valentiner,[1,11] Daniel Wicklein,[1,11,12] Hanna Maar,[1] Vera Labitzky,[1] Ann-Kristin Ahlers,[1] Sarah Starzonek,[1] Sandra Genduso,[1] Lisa Staffeldt,[1] Carolin Pahlow,[1] Anna-Maria Dück,[1] Christine Stürken,[1] Anke Baranowsky,[2] Alexander T. Bauer,[3] Etmar Bulk,[4] Albrecht Schwab,[4] Kristoffer Riecken,[5] Christian Börnchen,[6] Rainer Kiefmann,[6] Valsamma Abraham,[7] Horace M. DeLisser,[7] Timo Gemoll,[8] Jens K. Habermann,[8] Andreas Block,[9] Klaus Pantel,[10] and Udo Schumacher[1]

[1]Institute of Anatomy and Experimental Morphology, University Cancer Center Hamburg, University Medical Center Hamburg-Eppendorf, 20246 Hamburg, Germany; [2]Department of Trauma and Orthopedic Surgery, University Medical Center Hamburg-Eppendorf, 20246 Hamburg, Germany; [3]Department of Dermatology, University Cancer Center Hamburg, University Medical Center Hamburg-Eppendorf, 20246 Hamburg, Germany; [4]Institute of Physiology II, University of Münster, 48149 Münster, Germany; [5]Research Department Cell and Gene Therapy, Department of Stem Cell Transplantation, University Medical Center Hamburg-Eppendorf, 20246 Hamburg, Germany; [6]Department of Anesthesiology, University Medical Center Hamburg-Eppendorf, 20246 Hamburg, Germany; [7]Pulmonary, Allergy and Critical Care Division, Department of Medicine, School of Medicine, University of Pennsylvania, Philadelphia, PA 19104-4539, USA; [8]Section for Translational Surgical Oncology and Biobanking, Department of Surgery, University of Lübeck and University Medical Center Schleswig Holstein, Campus Lübeck, 23538 Lübeck, Germany; [9]Department of Oncology, University Cancer Center Hamburg, University Medical Center Hamburg-Eppendorf, 20246 Hamburg, Germany; [10]Institute of Tumor Biology, University Cancer Center Hamburg, University Medical Center Hamburg-Eppendorf, 20246 Hamburg, Germany

Extravasation of circulating tumor cells (CTCs) is critical for metastasis and is initiated by adhesive interactions between glycoligands on CTCs and E-selectin on endothelia. Here, we show that the clinically approved proteasome inhibitor bortezomib (BZM; Velcade) counteracts the cytokine-dependent induction of E-selectin in the lung mediated by the primary tumor, thereby impairing endothelial adhesion and thus spontaneous lung metastasis *in vivo*. However, the efficacy of BZM crucially depends on the tumor cells' E-selectin ligands, which determine distinct adhesion patterns. The canonical ligands sialyl-Lewis A (sLeA) and sLeX mediate particularly high-affinity E-selectin binding so that the incomplete E-selectin-reducing effect of BZM is not sufficient to disrupt adhesion or metastasis. In contrast, tumor cells lacking sLeA/X nevertheless bind E-selectin, but with low affinity, so that adhesion and lung metastasis are significantly diminished. Such low-affinity E-selectin ligands apparently consist of sialylated MGAT5 products on CD44. BZM no longer has anti-metastatic activity after CD44 knockdown in sLeA/X-negative tumor cells or E-selectin knockout in mice. sLeA/X can be determined by immunohistochemistry in cancer samples, which might aid patient stratification. These data suggest that BZM might act as a drug for inhibiting extravasation and thus distant metastasis formation in malignancies expressing low-affinity E-selectin ligands.

INTRODUCTION

The formation of distant metastases begins with the detachment of individual cancer cells or small cell clusters from the primary tumor (PT) and their invasion across the underlying basement membrane, migration through the adjacent extracellular matrix (ECM), and subsequent intravasation into the bloodstream as circulating tumor cells (CTCs).[1] During circulation, CTCs are exposed to different intrinsic and extrinsic selection pressures (anoikis induced by detachment from the ECM of the PT, mechanical shear stress, immune cells, especially natural killer [NK] cells).[2,3] Therefore, it is widely assumed that the extravasation of CTCs into the stroma of the organ of the later metastasis represents a bottleneck step of the metastatic cascade.[4,5] The initial event of extravasation is the attachment of CTCs to the inner lining of the blood vessels, the endothelium. In a close analogy to the adhesion cascade of leukocytes in the context of inflammation, this process is controlled by endothelial selection (E-selectin) and other cell adhesion molecules (CAMs).[5] Also, based on leukocyte adhesion, tumor cells seem to use very similar ligands for binding to E-selectin,[6,7] so the relevant literature summarizes that all E-selectin ligands on tumor cells contain the sialyl-Lewis A or X (sLeA/X) glyco-epitopes.[5] However, neither the full repertoire of possible E-selectin ligands on human tumor cells nor the role of other endothelial CAMs is fully known to date.[6,8] Several

Received 21 October 2021; accepted 10 January 2022;
https://doi.org/10.1016/j.ymthe.2022.01.017.

[11]These authors contributed equally

[12]Present address: Prof. Daniel Wicklein, Department of Anatomy and Cell Biology, University of Marburg, 35037 Marburg, Germany.

Correspondence: Tobias Lange, Institute of Anatomy and Experimental Morphology, University Cancer Center Hamburg, University Medical Center Hamburg-Eppendorf, 20246 Hamburg, Germany.
E-mail: to.lange@uke.de

 © 2022 The Author(s).
This is an open access article under the CC BY license (http://creativecommons.org/licenses/by/4.0/).

glycoconjugates have been considered to mediate E-selectin-, ICAM-1-, or VCAM-1 binding such as sLeA/X,[7] gangliosides,[9] and integrin $\alpha_{L/M}/\beta_2$ or integrin α_4/β_1,[10] respectively. The glycoproteins carrying sLeX and sLeA are also diverse, including PSGL-1, ESL-1, CD24, CD43, CD44, LGALS3BP, etc.[5] Given this diversity, it seems more logical to target the more defined groups of selectins and CAMs on the endothelium to impair CTC adhesion and thus extravasation and metastasis formation. In this regard, we previously reported that the genetic knock out of E-selectins in mice drastically impairs spontaneous metastasis formation in xenograft models of different human tumor types.[11–15] Therefore, we hypothesized that the pharmacologic inhibition of E-selectin expression should have anti-metastatic potential as well.

The regulation of E-selectin expression differs depending on the respective organ. For instance, E-selectin is transcriptionally induced by pro-inflammatory stimuli in the lung,[16] which ensures the enhanced extravasation of leukocytes into inflamed tissues. In contrast, E-selectin is constitutively expressed on bone marrow endothelium to enable lymphocyte trafficking under physiologic conditions.[17] In the context of cancer, several studies suggest that the PT itself promotes a systemic pro-inflammatory environment, which contributes to pre-metastatic niche formation at distant sites.[18] One well-described example is the focal induction of E-selectin on pulmonary endothelial cells (ECs) induced by PT-released soluble factors in the pre-metastatic phase. Metastatic tumor cells are directed to such foci of increased E-selectin expression in the lung.[19] Accordingly, the inhibition of cytokines such as tumor necrosis factor alpha (TNF-α) or interleukin (IL)-1 reduces metastatic spread to the lung *in vivo*,[20] and elevated cytokine serum levels indicate an unfavorable outcome of cancer patients.[21]

The pro-inflammatory cytokine stimulus mediates phosphorylation and ubiquitination of IκB, an inhibitory component of the nuclear factor κB (NF-κB) complex, which is then cleaved from the complex within the proteasome. Thereby, activated NF-κB translocates to the nucleus and induces expression of E-selectin and other CAMs of the leukocyte adhesion cascade.[22] The first clinically approved proteasome inhibitor was bortezomib (BZM; Velcade, formerly PS-341), which has been used for the treatment of multiple-myeloma patients over the last 15 years. BZM is known for its anti-proliferative, anti-angiogenic, and pro-apoptotic effects.[23] However, being an inhibitor of the proteasome, BZM should also reduce the cytokine-mediated induction of E-selectin and other CAMs. We therefore hypothesized that BZM might have anti-adhesive and thus anti-metastatic efficacy on solid human tumors. Such efficacy could be beneficial for patients with invasive tumors, particularly during transient periods of increased CTC and cytokine release. Such periods occur during medical interventions in patients with solid tumors such as biopsy and surgery.[24–26]

RESULTS

BZM impairs cytokine-mediated induction of E-selectin and other endothelial CAMs but has anti-adhesive efficacy on sLeA/X-negative tumor cells only

To test our hypothesis, we first investigated whether the pre-treatment of human umbilical vein ECs (HUVECs) with BZM counteracts cytokine-mediated upregulation of E-selectin and other CAMs. BZM significantly impaired IL-1α-mediated induction of E-selectin, ICAM-1, and VCAM-1, as determined by qPCR (Figure 1A) and flow cytometry (Figure 1B). Similar effects were observed with primary human pulmonary microvascular ECs (HPMECs) and after stimulation with TNF-α (Figure S3A).

Next, we analyzed whether the BZM-mediated reduction of E-selectin, ICAM-1, and VCAM-1 has a functional consequence for the adhesion of human tumor cells to HUVECs in laminar flow adhesion assays. In such assays, we distinguished firm adhesion from two types of loose adhesion, i.e., rolling and tethering (see illustration in Figure 1C). In our proof-of-concept experiments, we found that two gastrointestinal adenocarcinoma cell lines (HT29 and PaCa5061) were not impaired during endothelial adhesion upon BZM treatment of the ECs (BZM-resistant), while the adhesion of two tumor cell lines of non-epithelial origin (HOS and MeWo) was significantly reduced (BZM-sensitive; Figure 1D). Therefore, we hypothesized that the two subsets might differ in their ligands used for endothelial adhesion. In fact, both BZM-resistant cells showed notable levels of the canonical E-selectin ligands sLeA and sLeX, while both BZM-sensitive cells were sLeA/X-negative (Figure 1E). Due to the lack of sLeA/X expression in the case of BZM-sensitive cells, it remained to be demonstrated whether both subsets actually depend on E-selectin for endothelial adhesion. Using a validated E-selectin-blocking antibody, we observed that both subsets depended significantly (but to variable extents) on E-selectin during endothelial adhesion (Figure 1F). This finding indicated that sLeA/X-negative tumor cells can interact with E-selectin. To further corroborate this observation, we coated the microfluidic chambers with immobilized recombinant human (rh)E-selectin instead of with ECs and observed that the sLeA/X-negative cells were indeed able to develop adhesions on rhE-selectin under shear force conditions. Interestingly, sLeA/X-positive cells developed firm, flow-resistant adhesions on rhE-selectin, whereas sLeA/X-negative cells showed loose adhesive events in terms of rolling and tethering (Figure 1G). To test whether the two subsets were able to bind rhE-selectin under static conditions (in the absence of shear force), we incubated the tumor cells with fluorescence-labeled rhE-selectin and quantified E-selectin binding by flow cytometry. Importantly, sLeA/X-positive cells bound rhE-selectin while sLeA/X-negative cells did not (Figure 1H).

By using eight further human tumor cell lines (four sLeA/X-positive and four sLeA/X-negative), we confirmed that the sLeA/X status of the tumor cells was consistently associated with the anti-adhesive efficacy of BZM, the adhesion strength on rhE-selectin under flow, and the ability to bind rhE-selectin in the absence of shear force (Figure S1). All tested cell lines depended on E-selectin for adhesion on ECs (Figure S1). Of note, the myeloma cell lines AMO-1 and IM-9 displayed no cell surface sLeA/X expression and thus showed BZM-sensitive adhesion (Figure S1).

The anti-adhesive effect of BZM *in vitro* correlates with anti-metastatic efficacy of BZM *in vivo*

Given the partial anti-adhesive potential of BZM *in vitro*, we next investigated whether BZM reduces the spontaneous metastasis

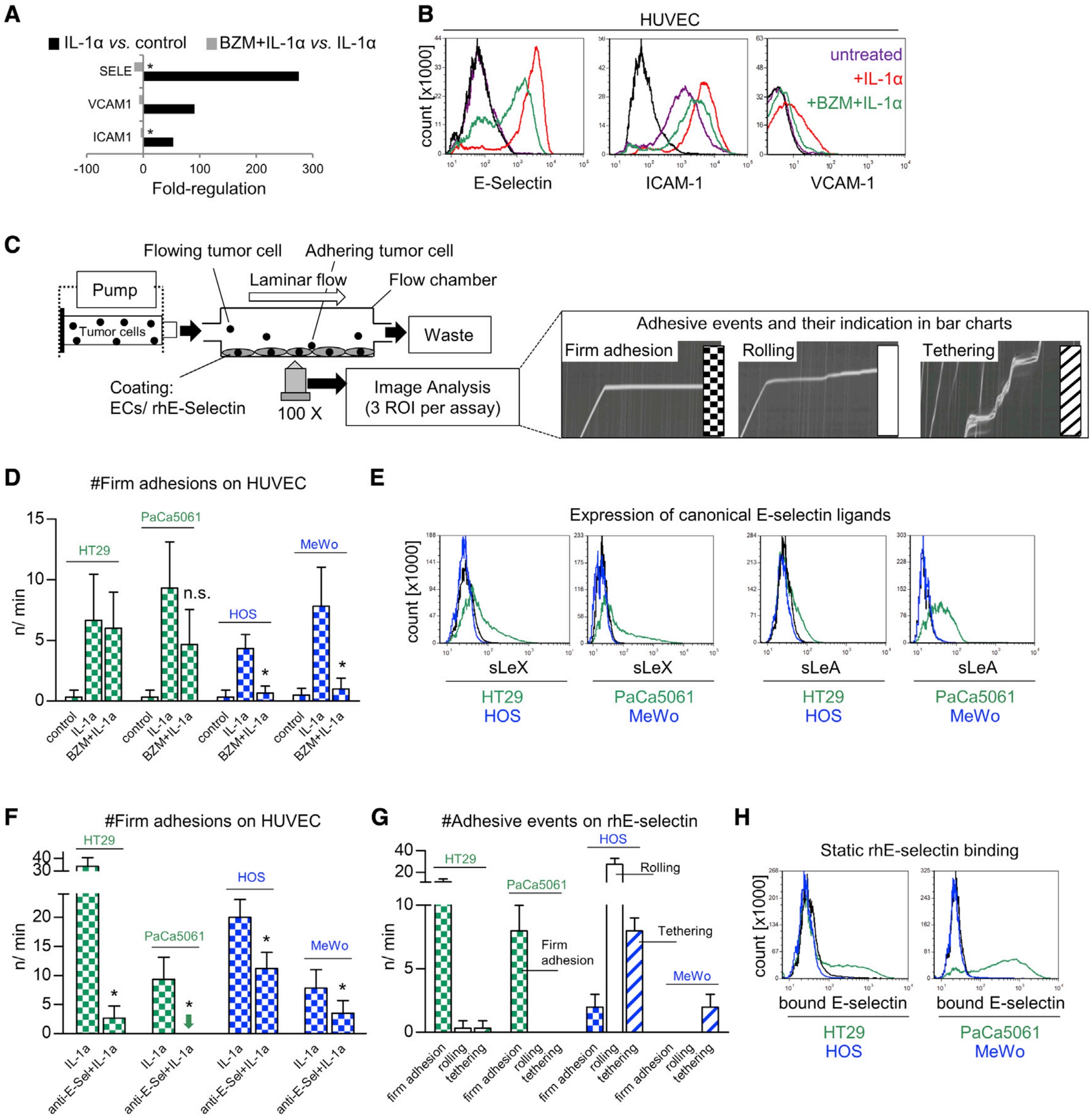

Figure 1. Bortezomib counteracts cytokine-mediated upregulation of E-selectin, ICAM-1, and VCAM-1, but its anti-adhesive efficacy depends on the adhesive properties of the tumor cells

(A and B) E-selectin, ICAM-1, and VCAM-1 gene expression (A) and protein levels (B) in/on human umbilical vein endothelial cells (HUVEC) treated ± IL-1α, ± BZM. (C) Laminar flow adhesion assays were performed as illustrated to measure the anti-adhesive potential of BZM. (D) Number of flow-resistant adhesions of indicated tumor cell lines on ECs treated ± IL-1α, ± BZM. (E) Tumor-cell-surface expression levels of sialyl-Lewis A (sLeA) and sLeX. (F) Number of flow-resistant adhesions of indicated tumor cell lines on ECs treated with IL-1α ± E-selectin-blocking antibody. (G) Number and quality of adhesive events (legend in C) of indicated tumor cell lines on immobilized rhE-selectin under laminar flow conditions. (H) Tumor-cell-surface E-selectin binding capacity under static conditions. See Figure S1 for verification of these observations with multiple human tumor cell lines. Bar charts represent mean ± SD of n = 3; black lines in histograms represent isotype controls (B and E) and binding of human IgG_1-Fc (H); *p < 0.05; n.s., not significant. Green indicates sLeA/X-positive tumor cell lines/models with BZM-resistant adhesion/metastasis, and blue indicates sLeA/X-negative tumor cell lines/models with BZM-sensitive adhesion/metastasis in all figures. Checked bars represent firm (flow-resistant) adhesions, open bars rolling adhesions, and striped bars tethering adhesions in all figures.

formation of solid human xenograft tumors *in vivo*. For this purpose, we injected HT29, PaCa5061 (both showing BZM-resistant adhesion), HOS, and MeWo cells (both showing BZM-sensitive adhesion) subcutaneously (s.c.) between the scapulae of immunodeficient mice and applied BZM or PBS intraperitoneally twice a week throughout the PT growth period (n = 10). The mice were sacrificed when PTs were ~1.5 cm^3 and the number of spontaneous lung and bone marrow metastases was determined (see illustration in Figure 2).

The PT growth period and the resulting tumor weight were largely unaltered by the BZM treatment (Figure 2A). Correspondingly, we observed no effects of BZM on xenograft PT cell proliferation (Ki67), apoptosis (pH2AX), or angiogenesis (mCD31) as compared with in the PBS treatment (Figures S2A–S2C). However, despite the lack of effects on the PTs, the pulmonary metastatic cell load was significantly reduced upon BZM treatment in the case of xenograft models that were derived from the sLeA/X-negative cells (HOS and MeWo) that had shown the BZM-sensitive adhesion pattern *in vitro* (Figure 2B). In the HOS model, lung metastases were even reduced, although the PT growth period was slightly prolonged (#$p < 0.01$, analysis of covariance [ANCOVA] including the growth period as covariate; Figure 2B). In contrast, BZM did not reduce the lung metastasis formation of xenografts derived from sLeA/X-positive cells (HT29 and PaCa5061), which had shown the BZM-resistant adhesion pattern *in vitro* (Figure 2B). Based on qPCR analyses, we verified that the applied BZM treatment significantly reduced *Sele* (encoding E-selectin), *Icam1*, and *Vcam1* expression in the lungs of xenograft PT-bearing mice irrespective of whether or not BZM had anti-metastatic activity (Figure 2C).

Using primary murine pulmonary ECs (MPECs[27]), we confirmed that BZM counteracts the cytokine-mediated upregulation of E-selectin at the protein level also in mice (Figure 2D). Furthermore, we observed the same partial anti-adhesive efficacy of BZM in laminar flow adhesion assays, as determined before on human ECs (Figure 2D). In addition, we employed a murine *ex vivo* lung perfusion model combined with real-time epifluorescence video microscopy (setup illustrated in Figure 2E[28]). Thereby, we validated that a systemic cytokine stimulus facilitates the active, flow-resistant adhesion of sLeA/X-negative HOS cells to the vessel wall of pulmonary precapillary arterioles. The diameters of such microvessels were approximately twice as large as the diameters of the adhering HOS cells. When we applied BZM prior to the cytokine stimulus, the HOS cells got stuck in capillaries with a diameter similar to that of the tumor cells (Figure 2E). This observation indicated that the BZM pre-treatment prevented the active adhesion of sLeA/X-negative human tumor cells to the murine lung microvasculature *in situ*. Furthermore, we excluded that the reduced pulmonary metastatic cell load upon BZM treatment was due to impaired metastatic outgrowth (colonization) by counting the number of tumor cells per lung metastasis in histological sections. There was no difference in the average number of tumor cells per lung metastasis in the PBS- versus BZM-treated mice in both BZM-sensitive models (Figure S2D). Finally, BZM had no significant effect on bone metastasis (Figure S2E), even in the BZM-sensitive models, which is in accordance with the aforementioned constitutive, but not cytokine-regulated E-selectin expression in the bone marrow. These findings collectively demonstrated that the anti-metastatic effect of BZM, if present, was most likely due to its anti-adhesive activity.

The anti-adhesive activity of BZM is due to the loss of E-selectin

BZM treatment significantly reduced not only *Sele*, but also *Icam1* and *Vcam1*, expression in the lungs of s.c. xenograft tumor-bearing mice (Figure 2C) and of E-selectin, ICAM-1, and VCAM-1 protein levels on ECs *in vitro* (Figure 1B). In addition, flow adhesion assays with validated blocking antibodies against ICAM-1 and VCAM-1 demonstrated that ICAM-1 mediates loose adhesions of PaCa5061 (rolling) and MeWo (rolling and tethering) cells on ECs (Figure 3A). More importantly, however, firm adhesions of both BZM-sensitive cells (HOS and MeWo) were reduced upon VCAM-1 blockade (Figure 3B). Based on this observation, it was critically important to analyze whether the partial anti-adhesive activity of BZM was due to the loss of E-selectin alone or of ICAM-1 or VCAM-1 as well. For this purpose, we generated a HUVEC subline with short hairpin RNA (shRNA)-mediated, stable depletion of E-selectin. These derivatives showed drastically less E-selectin induction upon IL-1α, while the effects of IL-1α ± BZM on ICAM-1 and VCAM-1 were largely unaffected (Figure 3C). Importantly, the two sLeA/X-positive (BZM-resistant) cell lines showed no firm adhesions on E-selectin-depleted HUVECs anymore (Figure 3D), reflecting the striking effect of the E-selectin-blocking antibody in the case of these cells (Figure 1F). In contrast, the two sLeA/X-negative (BZM-sensitive) cell lines developed a reduced, but clearly detectable, number of adhesions on E-selectin-depleted HUVECs per minute (Figure 3D), reflecting the less striking effect of the E-selectin blocking antibody in the case of these cells (Figure 1F). However, these adhesions were not further reduced by pre-treating the ECs with BZM (Figure 3D). Thus, the "remaining" (presumably VCAM-1-mediated) adhesions on E-selectin-depleted HUVECs were not impaired by the BZM-mediated loss of VCAM-1 expression (Figure 3C). In a proof-of-principle experiment with one BZM-sensitive xenograft model (3R principle), we observed that BZM "loses" its anti-metastatic effect on HOS xenografts in *Sele*$^{-/-}$ mice (Figure 3E), while its moderate effect on the HOS xenograft tumor growth (slightly prolonged growth period) known from E-selectin wild-type mice (Figure 2A) was again detectable (Figure 3E). This effect on the PT was considered a covariate when the potential difference in lung metastasis numbers was calculated (ANCOVA).

Tumor cells with BZM-sensitive adhesion show weak endothelial adhesion strength

All tested cell lines depended (to variable extents) on E-selectin for endothelial adhesion (Figures 1D and S1), and E-selectin was responsible for the anti-adhesive and anti-metastatic effects of BZM (Figures 3D and 3E). However, only sLeA/X-negative tumor cells showed impaired endothelial adhesions when the ECs were treated with BZM (Figures 1C, 1F, and S1). As shown above, these BZM-sensitive cells developed rather loose adhesions on rhE-selectin under flow and were unable to bind rhE-selectin in the absence of shear force. Therefore, we hypothesized that the endothelial adhesions of the BZM-sensitive cells might be less stable than those of BZM-resistant cells so that, in their case, the incomplete BZM effect on E-selectin (~60% reduction) was sufficient to reduce the number of firm adhesions and thus lung metastasis. To test this hypothesis, we next assessed the stability of the endothelial adhesions formed by BZM-sensitive versus -resistant cells. Interestingly, most of the endothelial adhesions of sLeA/X-negative (BZM-sensitive) tumor cells established at the

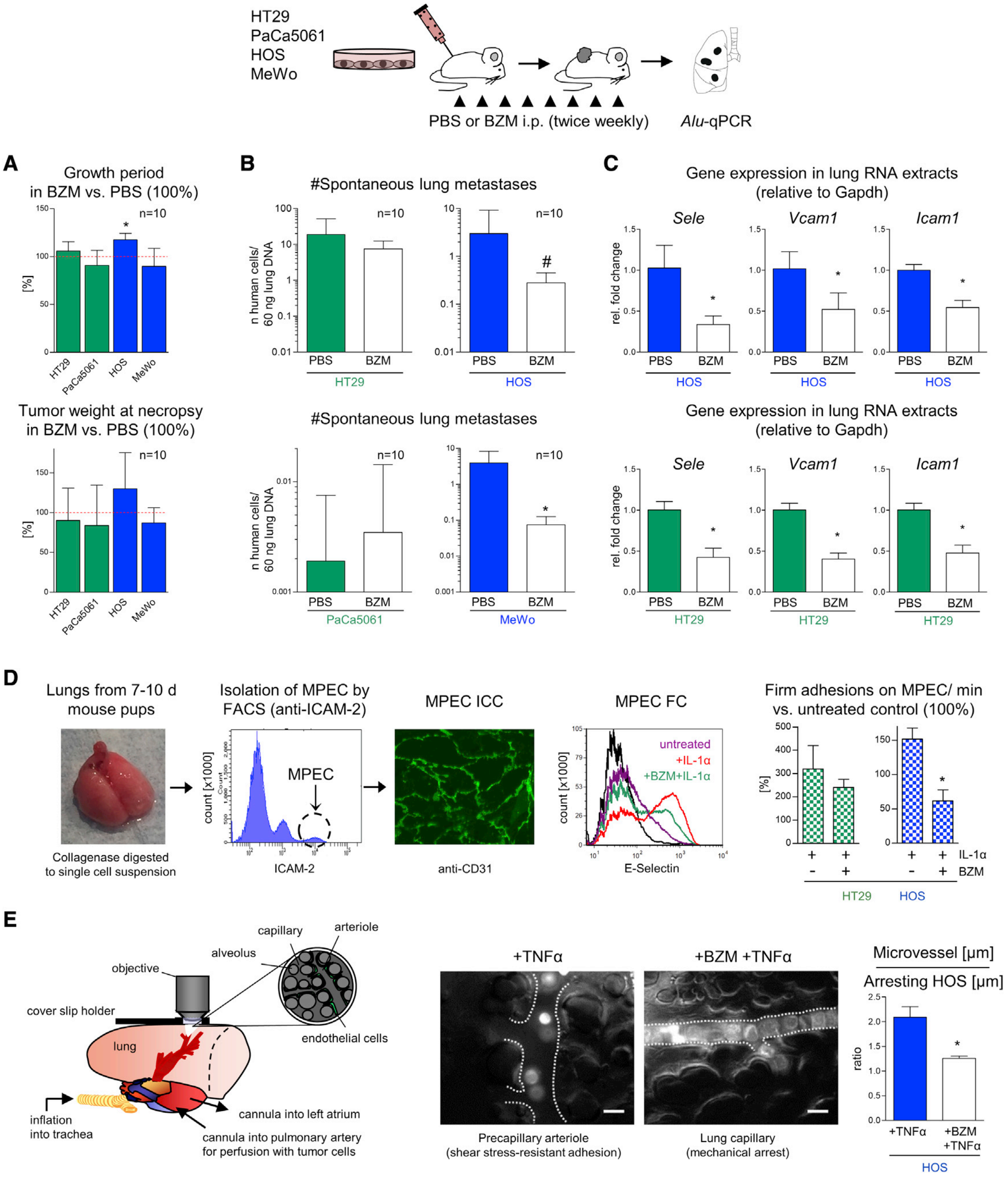

Figure 2. The anti-adhesive efficacy of BZM *in vitro* correlates with anti-metastatic efficacy *in vivo*

(A and B) Percentage change of subcutaneous primary tumor growth periods and tumor weights (A) as well as absolute numbers of spontaneous lung metastases (B) at necropsy of indicated human tumor cell line xenografts treated with PBS (control = 100% in A) or BZM as illustrated in the scheme. The minor effect on the tumor growth period upon BZM treatment (HOS) was considered as a covariate in the statistical analysis of lung metastasis numbers (ANCOVA, #$p < 0.01$, B). (C) Pulmonary *Sele* (encoding E-selectin), *Vcam1*, and *Icam1* expression levels in PBS- and BZM-treated xenograft tumor-bearing mice. (D) Isolation of ICAM-2-positive, CD31-positive murine pulmonary ECs (MPECs), E-selectin expression after IL-1α ± BZM treatment, and number of flow-resistant adhesions of the indicated cell lines on MPECs. (E) Adhesive behavior of human tumor cells in different segments of the murine pulmonary microcirculation after cytokine stimulation ± BZM pre-treatment (*ex vivo* lung perfusion model). Bar charts represent mean ± SD; scale bars in (E) represent 25 μm; *$p < 0.05$. See Figure S2 for potential off-target effects of BZM *in vivo* (primary tumors, metastatic outgrowth, and bone marrow metastasis). FACS, fluorescence-activated cell sorting; ICC, immunocytochemistry; FC, flow cytometry.

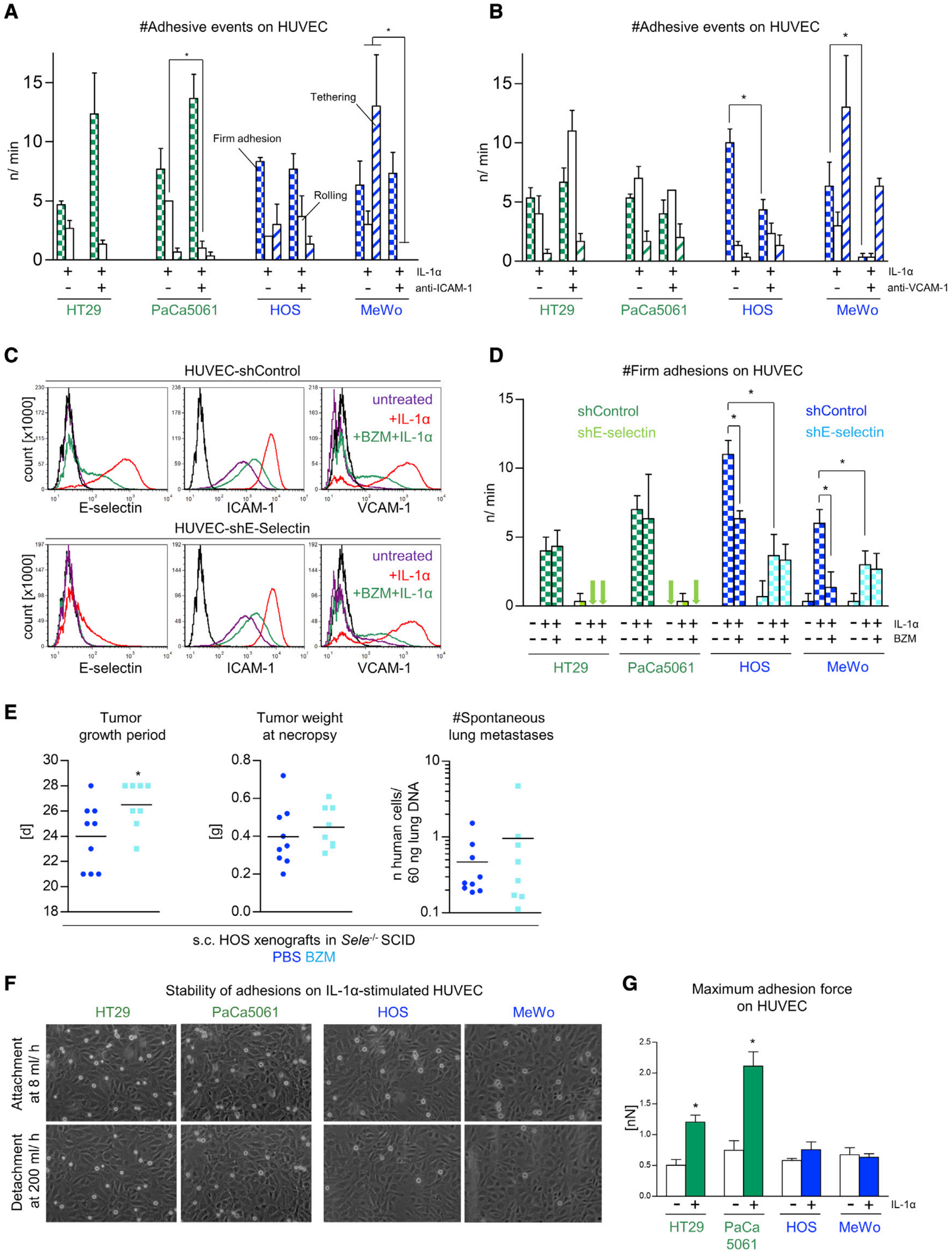

Figure 3. The anti-adhesive and anti-metastatic effects of BZM depend on E-selectin, and the endothelial adhesion strength depends on the tumor cells' E-selectin ligand statuses

(A and B) Number and quality of dynamic adhesions of indicated tumor cell lines on ECs after cytokine-stimulation ± ICAM-1 (A) or VCAM-1 blockade (B). (C) E-selectin, ICAM-1, and VCAM-1 cell-surface protein expression on ECs (treated ± IL-1α ± BZM) after stable (shRNA-mediated) depletion of E-selectin or control transduction. (D) Number of flow-resistant adhesions of indicated tumor cell lines on control or E-selectin-depleted ECs, treated ± IL-1α ± BZM. (E) s.c. HOS xenograft primary tumor growth

(legend continued on next page)

physiological flow rate (8 mL/h) could be broken by increasing the laminar flow to a maximum (200 mL/h). In contrast, the adhesions of sLeA/X-positive (BZM-resistant) tumor cells were largely non-detachable (Figure 3F). To quantify the tumor-endothelium-binding stability more precisely, we additionally applied single-cell force spectroscopy to determine the maximum adhesion force required for retracting tumor cells from ECs. Interestingly, the maximum adhesion force strongly increased upon cytokine stimulation of the ECs in the case of sLeA/X-positive, but not sLeA/X-negative, cells (Figure 3G).

Endothelial adhesion of BZM-resistant versus -sensitive tumor cells is mediated by distinct classes of pro-adhesive glycans

Next, we aimed to characterize the different E-selectin ligands determining the anti-adhesive and anti-metastatic efficacy of BZM in further detail. Briefly, pre-treatment of the tumor cells with neuraminidase (ND) notably reduced endothelial adhesion of all tested cell lines (Figure 4A), suggesting a still-important role of sialic acid for EC binding also in the case of sLeA/X-negative tumor cells. Accordingly, both cell lines showed detectable levels of cell surface α-2,3-sialic acid (determined by *Maackia amurensis* lectin II [MAA-II] using flow cytometry), while HOS, but not MeWo, cells additionally carried α-2,6-sialic acid residues (determined by *Sambucus nigra* (SNA-I) lectin). ND treatment specifically reduced α-2,3-sialic acid residues but not α-2,6 sialic residues (Figure S3B). In the case of sLeA/X-positive cells, ND treatment also reduced sLeA expression and static rhE-selectin binding (Figure 4A, histograms). Inhibition of *O*-GalNAc-glycosylation with GalNAc-α-*O*-benzyl (GOB) reduced the endothelial adhesion of HT29 and PaCa5061 cells (accompanied by decreased sLeA expression and static rhE-selectin binding), while the adhesion of HOS and MeWo cells remained unaltered (Figure 4B). Vice versa, the inhibition of *N*-glycosylation with swainsonine (Sw) reduced the adhesion of HOS and MeWo cells but did not impair the adhesion of HT29 and PaCa5061 cells (Figure 4C). In the case of HT29 cells, inhibition of *N*-glycosylation even improved endothelial adhesion (Figure 4C). The efficacy of the used Sw treatment protocol (inhibition of *N*-glycosylation in the Golgi) was verified by demonstrating decreased cell surface poly-*N*-acetyllactosamine (poly-LacNAc) (detected by the binding of *Datura stramonium* lectin [DSL]) and β-1,6-GlcNAc-branches (detected by the binding of *Phaseolus vulgaris* leukoagglutinin [PHA-L]) (Figure 4C). β-1,6-GlcNAc-branches are commonly expressed on tumor cells and represent *N*-linked scaffolds for poly-LacNAc-chains.[29] Non-specific cleavage of cell-surface glycoproteins using pronase remarkably reduced the endothelial adhesion of HOS and MeWo but not of HT29 and PaCa5061 cells (Figure 4D). Concerning sLeA expression and static rhE-selectin binding, only PaCa5061 cells showed a partial reduction of sLeA expression upon pronase treatment (Figure 4D).

Taken together, sLeA/X-positive tumor cells with the BZM-resistant adhesion pattern (HT29 and PaCa5061) mainly depended on *O*-GalNAc-glycosylation, while sLeA/X-negative tumor cells with the BZM-sensitive adhesion (HOS and MeWo) mainly depended on *N*-glycosylation for adhesion. Given this clear dichotomy, we next profiled glycosyltransferase expression levels in HOS and MeWo cells each in comparison to HT29 cells. By this approach, we determined a strikingly decreased expression of the core 2 *O*-glycan synthase C2GNT2 (GCNT3) in both sLeA/X-negative cells (Figures 4E and 4F; see Table S1 for the entire profiler dataset). Therefore, we investigated whether sLeA/X synthesis can be impaired by shRNA-mediated depletion of C2GNT2. Intriguingly, sLeA expression and static rhE-selectin binding capacity were notably reduced upon C2GNT2 depletion in HT29 cells (Figure 4G) while sLeX was increased. The inserted box in Figure 4 illustrates key glycosylation steps and targets of the chosen inhibitors/knockdowns relevant to this study.

CD44 is a major carrier of non-canonical E-selectin ligands

Next, we aimed to determine the glycoprotein carrier(s) of non-canonical E-selectin ligands in the case of the BZM-sensitive cell lines (HOS and MeWo), both of which depended on glycoprotein-bound *N*-glycans for adhesion, as determined by the pronase and Sw treatments. Based on the literature, CD44, CD24, ESL-1, PSGL-1, CD43, MUC1, and LGALS3BP are the most commonly described glycoprotein carriers of E-selectin ligands.[5] Interestingly, HOS and MeWo displayed particularly strong expressions of CD44 but rather weak (HOS) or even no (MeWo) expression of CD24 (in comparison to HT29 and PaCa5061 cells; Figure 5A), while CD43, ESL-1, LGALS3BP, MUC-1, and PSGL-1 were not detectable on these cell lines (data not shown). Therefore, we stably depleted CD44 in HOS and MeWo cells by shRNA (Figure 5B), which abrogated their adhesiveness on ECs under flow conditions (Figure 5B). Consistently, PNGaseF and ND treatment of HOS and MeWo protein extracts resulted in downward mobility shifts of CD44 in western blot (WB) analyses (Figure 5C). Likewise, pre-treating the cells with Sw prior to protein extraction also led to a detectable downward mobility shift of CD44 (Figure 5C). These findings demonstrated that CD44 of both cell lines was sialylated and *N*-glycosylated. To further address the question of whether normal *N*-glycan maturation adds β-1,6-GlcNAc-branches and poly-LacNAc chains (both of which were reduced on the less adhesive HOS and MeWo cells after Sw treatment; Figure 4C) specifically to CD44 in the case of these two cell lines, similar amounts of protein extracts from Sw-treated and control cells were incubated with PHA-L agarose (binding to β-1,6-GlcNAc-branches[30]) or DSL-agarose (binding to poly-LacNAc[31]). Interestingly, by this lectin pull down approach, we could precipitate notable amounts of CD44 with both lectins from both cell lines, and we observed significant reductions of precipitated CD44 after Sw treatment (Figure 5D). Golgi β1,6-N-acetylglucosaminyltransferase V

periods and tumor weights as well as spontaneous lung metastasis numbers at necropsy in PBS- (control) or BZM-treated *Sele*$^{-/-}$ SCID mice. This treatment was carried out as in Figures 2A and 2B. The effect on the tumor growth period was considered as a covariate in the statistical analysis of lung metastasis numbers (ANCOVA). (F) Stability of endothelial adhesions of indicated tumor cell lines on cytokine-stimulated ECs. (G) Maximum adhesion force required for retracting single tumor cells of the indicated cell lines from control versus cytokine-stimulated ECs. Bar charts represent mean ± SD of n = 3 (A, B, and D) and n ≥ 3 (G); *p < 0.05.

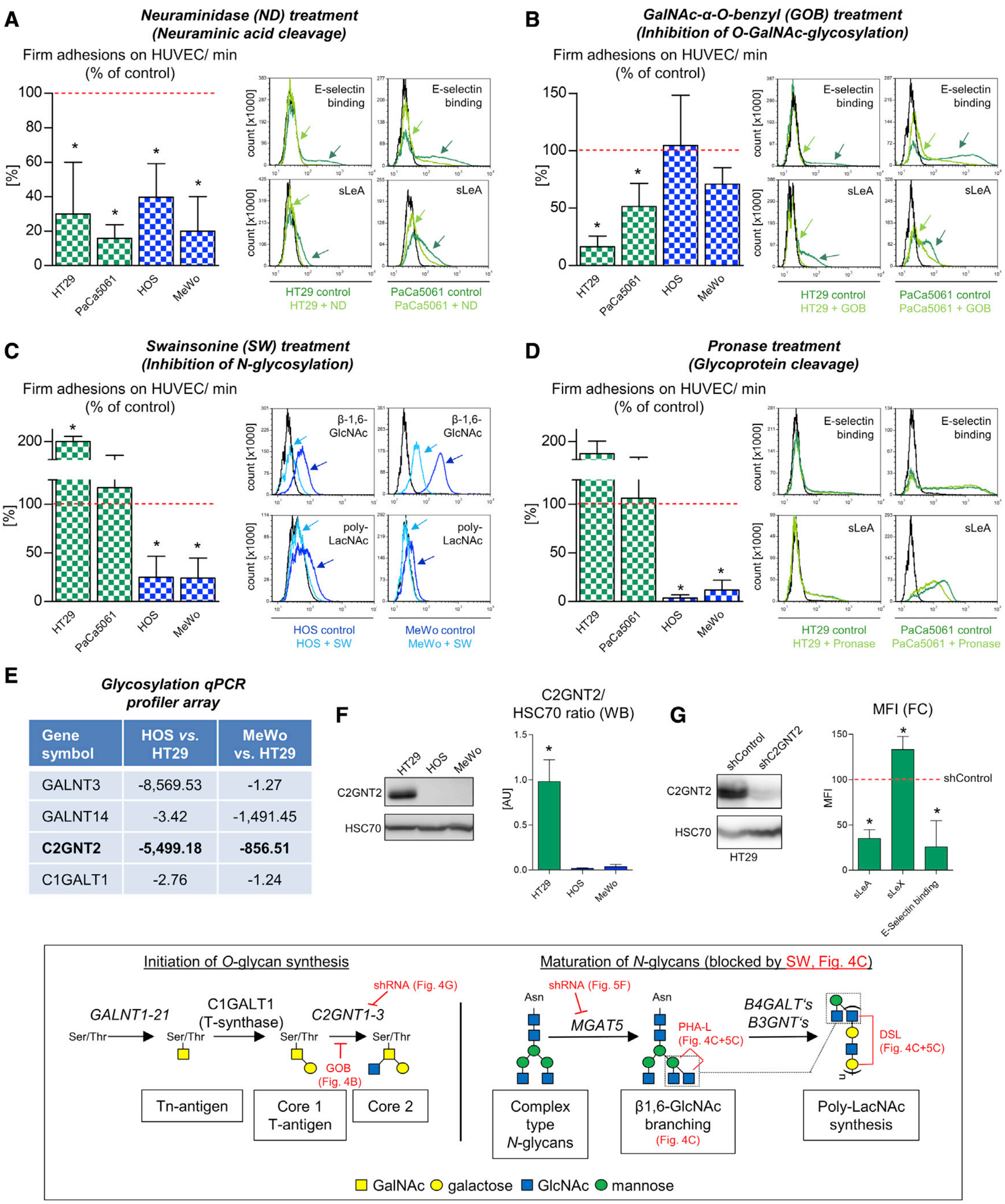

Gene symbol	HOS *vs.* HT29	MeWo vs. HT29
GALNT3	-8,569.53	-1.27
GALNT14	-3.42	-1,491.45
**C2GNT2**	**-5,499.18**	**-856.51**
C1GALT1	-2.76	-1.24

Figure 4. Pro-adhesive glycans on tumor cells with BZM-sensitive versus BZM-resistant adhesion

(A–D) Percentage changes of flow-resistant adhesions on cytokine-stimulated ECs as well as absolute changes in static E-selectin binding capacity and different tumor-cell-surface carbohydrate residue levels before and after indicated treatments of tumor cells from the different cell lines. (E) Human glycosyltransferase gene expression profiler array. (F) Differential protein levels of C2GNT2 in the indicated tumor cell lines. (G) Tumor-cell-surface sLeA/X expression and static E-selectin binding capacity after shRNA-mediated knock down of C2GNT2. The inserted box represents key glycosylation steps relevant to this study. Arrows in (A)–(D) highlight changes in E-selectin binding, sLeA expression, or β-1,6-GlcNAc (lectin PHA-L binding site) and poly-LacNAc (lectin DSL binding site) levels upon treatments. ND, neuraminidase; GOB, GalNAc-α-*O*-benzyl; SW, swainsonine; PHA-L, *Phaseolus vulgaris* leukoagglutinin; DSL, *Datura stramonium* lectin. Bar charts represent mean ± SD of three replicates; *p < 0.05

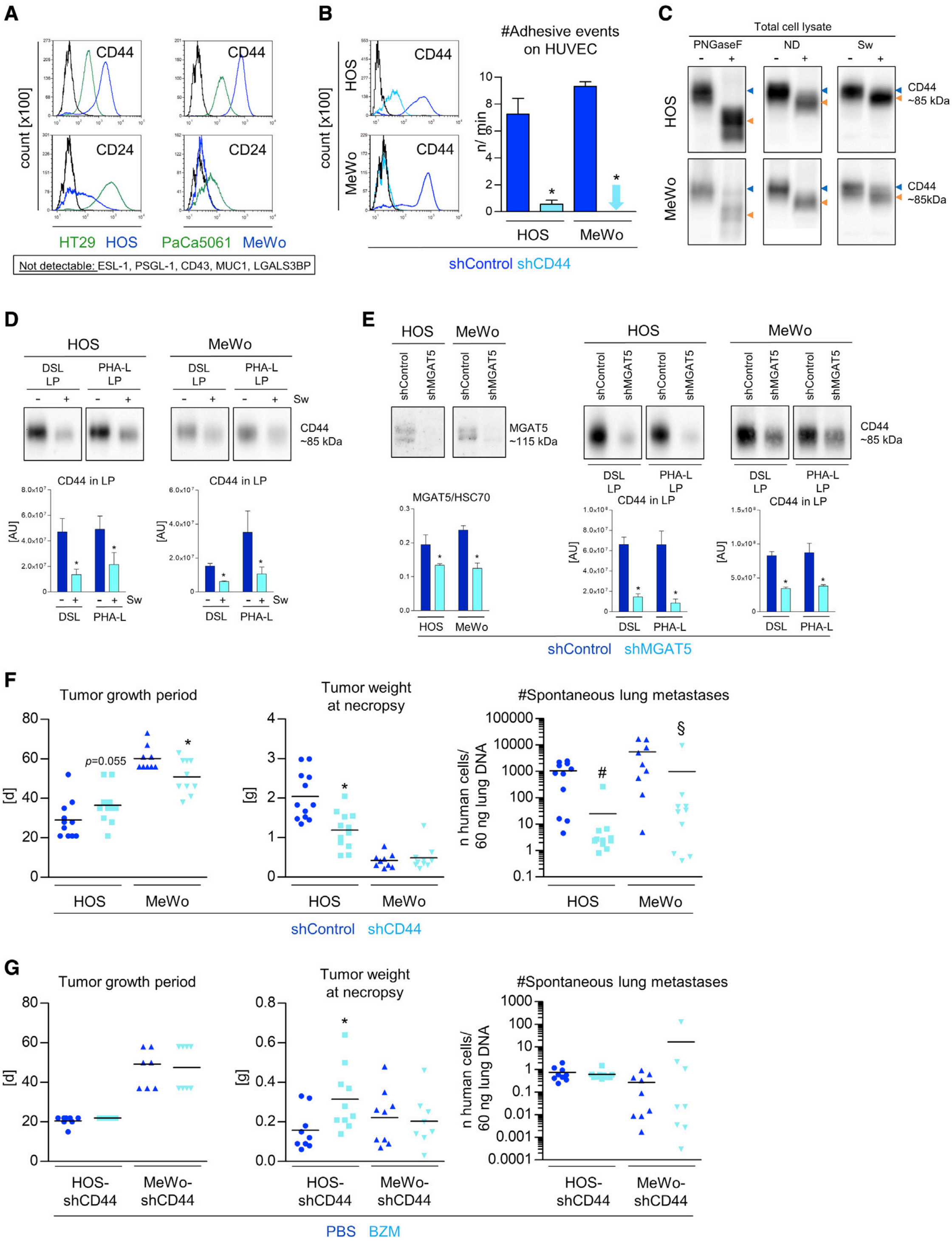

Figure 5. Pro-adhesive glycoproteins on tumor cells with BZM-sensitive adhesion

(A) Cell-surface CD24 and CD44 levels on the indicated tumor cell lines. (B) shRNA-mediated depletion of CD44 and numbers of adhesive events of control and CD44 knockdown tumor cells on ECs. (C) CD44 WB of total protein extracts from the annotated tumor cell lines. Peptide *N*-glycosidase F (PNGaseF) and neuraminidase (ND) were used to treat extracted proteins prior to WB; swainsonine (Sw) was used to treat tumor cells prior to protein extraction. (D) CD44 protein quantification in lectin precipitates (LPs) from protein extracts of tumor cells treated ± swainsonine. (E) Generation of control and MGAT5 knockdown derivatives of indicated cell lines and CD44 protein quantification in LPs pulled down from extracts of the respective derivative. (F) s.c. xenograft primary tumor growth periods and tumor weights as well as spontaneous lung

(legend continued on next page)

(MGAT5) is the key enzyme for the biosynthesis of β-1,6-GlcNAc-branched *N*-glycans. Therefore, we knocked down MGAT5 in HOS and MeWo cells (Figure 5E) and observed that much less CD44 could be precipitated with DSL- and PHA-L from cell extracts of MGAT5 knockdown cells (Figure 5E). These findings demonstrate that CD44 is a substrate of MGAT5 and carries β-1,6-GlcNAc-branches, presumably elongated by poly-LacNAc chains.

Furthermore, CD44 depletion abrogated the spontaneous pulmonary metastasis formation of HOS and MeWo xenografts *in vivo*. This effect was highly significant despite concurrent effects of the CD44 knockdown at the PT level (ANCOVA test including growth period [MeWo] and tumor weight [HOS] as covariates; Figure 5F). Ultimately, we observed that BZM again lose sits anti-metastatic effect on HOS and MeWo xenografts when CD44 is depleted (Figure 5G). Here, the BZM treatment slightly improved PT formation in the case of the HOS-shCD44 model, but lung metastasis was nevertheless unchanged (ANCOVA test including tumor weight as a covariate; Figure 5G).

sLeA and sLeX determine the efficacy of BZM in additional cell-line-based and patient-derived models

Next, we aimed to validate the apparent correlation between the anti-adhesive efficacy of BZM *in vitro* and its anti-metastatic efficacy *in vivo* by testing additional sLeA/X-positive versus sLeA/X-negative cells from the confirmatory experiments of Figure S1. We chose GC5023 gastric cancer and SW2 small-cell lung cancer cells as another sLeA/X-positive and sLeA/X-negative model, respectively. In accordance with the divergent anti-adhesive efficacy of BZM on these cell lines, we observed no anti-metastatic activity of BZM on GC5023 xenografts but did on SW2 xenografts (Figure 6A). The only detectable pulmonary metastasis phenotype of SW2 xenografts in both the PBS and BZM groups were single-cell metastases as determined by anti-neural cell adhesion molecule (NCAM) immunostainings (Figure S4A). Therefore, the reduced pulmonary metastatic burden upon BZM treatment was again not due to impaired metastatic colonization/outgrowth.

In addition, we chose SKOV3 ovarian cancer cells for spontaneous metastasis experiments *in vivo* as these cells also fulfilled all criteria of the BZM-sensitive tumor subset *in vitro* (Figure 6B). Unexpectedly, however, BZM had no anti-metastatic activity on SKOV3 xenografts *in vivo* (Figure 6C). Intriguingly, in contrast to *in-vitro*-cultured cells, *in-vivo*-grown SKOV3 xenograft tumors did show a considerable expression of sLeA (Figure 6D). In the view of the aforementioned data, this upregulation of sLeA *in vivo* might explain the switch from the BZM-sensitive behavior *in vitro* to the BZM-resistant behavior of the SKOV3 model *in vivo*. Such sLeA induction *in vivo* was not observed in the case of HOS, MeWo, and SW2 xenografts (Figure S4B).

Finally, we aimed to validate our cell-line-based observations with patient-derived models. We chose colorectal cancer (CRC) because sLeA (CA19-9) is a prognostic tumor marker in this disease.[32] In accordance with the cell-line data, the sLeA-positive CRC PT model PT1003 (see Supplemental information for further information) was capable of binding rhE-selectin under static conditions and adhered firmly on immobilized rhE-selectin under flow. It strongly depended on E-selectin for endothelial adhesion but was not impaired by the anti-adhesive BZM treatment (Figures 7A–7C). In contrast, the sLeA/X-negative CRC PT model PT457 did not bind rhE-selectin under static conditions and interacted only loosely with E-selectin under flow. Nevertheless, it depended on E-selectin for endothelial adhesion and was impaired by BZM (Figures 7A–7C). Different sLeA expression levels could be determined on primary patient material as exemplarily shown for colon and ovarian cancer surgical specimens (Figure S4C); therefore, patients could possibly be stratified for the anti-metastatic BZM treatment based on anti-sLeA immunohistochemistry (IHC) as a first step.

DISCUSSION

This study demonstrates that the clinically approved proteasome inhibitor BZM (Velcade) counteracts the induction of E-selectin expression in pulmonary microvessels in the presence of s.c. xenograft PTs in mice (by ∼60%). This incomplete inhibitory effect is sufficient to reduce the endothelial adhesion and spontaneous metastasis of tumor cells/xenografts lacking the canonical E-selectin ligands sLeA and sLeX but is obviously insufficient to reduce the adhesion and metastasis of sLeA/X-positive tumors that are characterized by marked E-selectin binding affinity.

These glycan epitopes are widely considered to be essential for E-selectin binding on human tumor cells.[5–7] However, we observed that antibody blockade and shRNA-mediated depletion of E-selectin significantly impairs the endothelial adhesion of sLeA/X-negative tumor cells as well. Moreover, such cells are able to roll and tether on isolated rhE-selectin under flow conditions. These findings demonstrate that sLeA/X-negative cells do interact with E-selectin so that "non-canonical" ligands (other than sLeA/X) apparently exist on human tumor cells. However, compared with sLeA/X-positive tumor cells, the E-selectin blockade and depletion effects are less striking and the dynamic adhesions on rhE-selectin and ECs are less stable in the case of sLeA/X-negative tumor cells. Additionally, sLeA/X-negative tumor cells are specifically unable to bind rhE-selectin under static conditions. Therefore, we conclude that the non-canonical ligands bind E-selectin with low affinity and are functional under

metastasis numbers at necropsy of shControl versus shCD44 derivatives of the indicated cell lines (F, $^{\#}p < 0.05$ considering tumor weight as a covariate; $^{\S}p < 0.05$ considering tumor growth period as a covariate, ANCOVA). (G) s.c. xenograft primary tumor growth periods and tumor weights as well as spontaneous lung metastasis numbers at necropsy of shCD44 derivatives of the indicated cell lines treated with PBS (control) or BZM. This treatment was carried out as in Figures 2A and 2B. The effect on the tumor weight (HOS-shCD44) was considered as a covariate in the statistical analysis of lung metastasis numbers (ANCOVA). Bar charts represent mean ± SD of three replicates; black lines in histograms represent isotype controls; black lines in scatterplots represent means; $^{*}p < 0.05$.

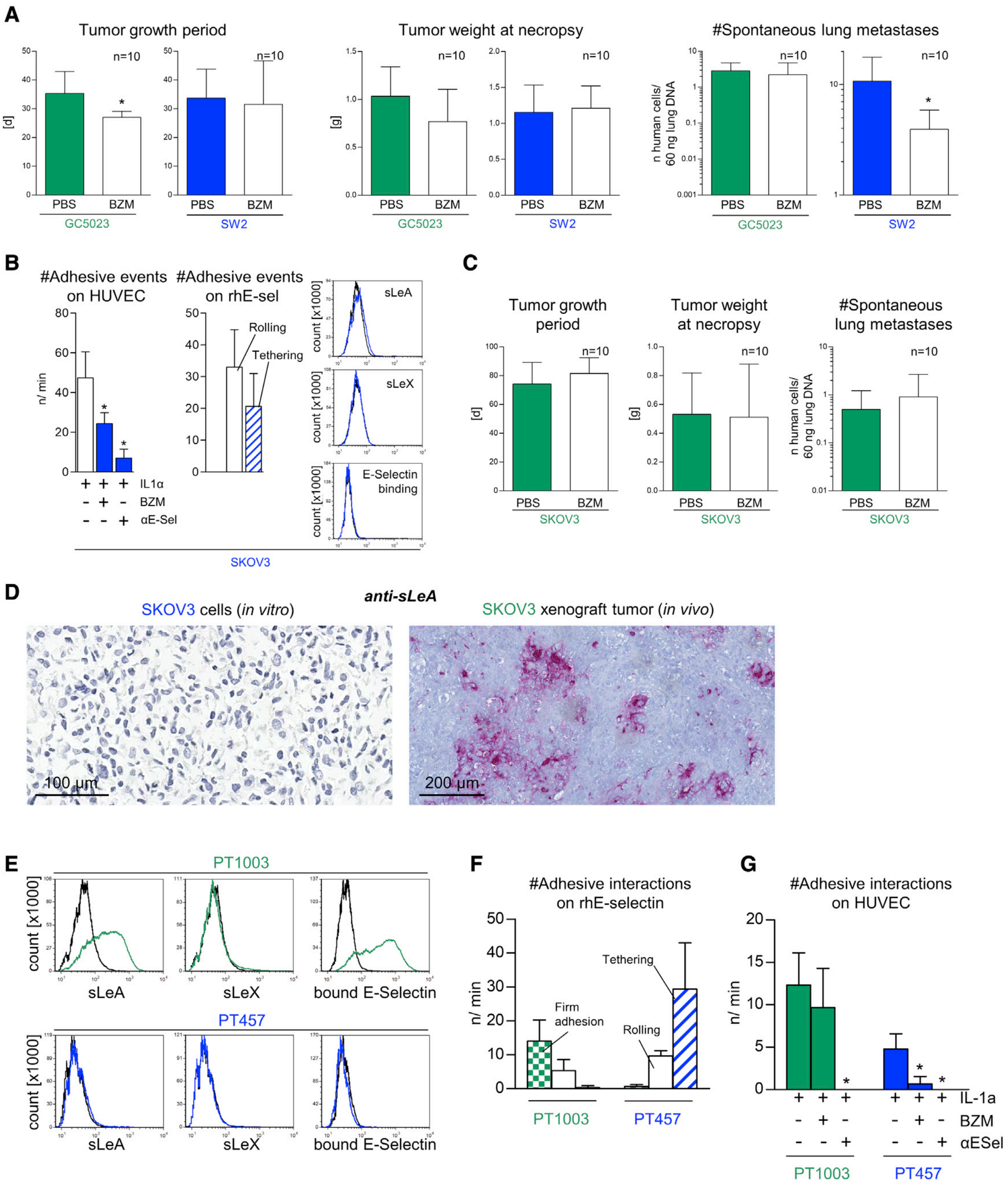

Figure 6. SLeA and/or sLeX determine the efficacy of BZM in additional cell-line-based and patient-derived, organoid-based models

(A) s.c. primary tumor growth periods and tumor weights as well as spontaneous lung metastasis numbers at necropsy of indicated human tumor cell line xenografts treated with PBS or BZM. This treatment was carried out as in Figures 2A and 2B. The minor effect on the tumor growth period upon BZM treatment (GC5023) was considered as a covariate in the statistical analysis of lung metastasis numbers (ANCOVA). (B) Number of adhesive events of SKOV3 cells on ECs treated ± IL1α ± BZM ± E-selectin-blocking antibody and on immobilized rhE-selectin. Representative histograms of tumor-cell-surface sLeA/X expression and static E-selectin binding capacity of SKOV3 cells. (C) s.c. primary tumor growth periods and tumor weights as well as spontaneous lung metastasis numbers at necropsy of SKOV3 xenografts treated with PBS or BZM. This treatment was carried out as in Figures 2A and 2B. (D) Anti-sLeA immunostainings of *in-vitro*-cultivated versus *in-vivo*-grown SKOV3 cells versus xenograft tumors, respectively. (E) Representative histograms of tumor-cell-surface sLeA/X expression and static E-selectin binding capacity on patient-derived, organoid-based colorectal cancer models as indicated. (F and G) Numbers of adhesive interactions of single-cell suspensions of these models on immobilized rhE-selectin (F) and ECs treated ± IL-1α ± BZM ± E-selectin-blocking antibody (G). Bar charts represent mean ± SD of n = 10 (A and C) or three replicates (B, F, and G); black lines in histograms represent isotype controls or binding of human IgG_1-Fc (C); *$p < 0.05$.

A

		Expression of		Static binding capacity	Dynamic binding capacity/ adhesion properties					Adhesion impaired by				
		sLeA	sLeX	rhE-Sel	rhE-Sel	Adh force	E-Sel block	VCAM-1 block	Adh on HUVEC-shE-Sel	ND	GOB	SW	Pron	sh CD44
BZM-resistant	HT29	+	+	+	firm	++	+++	-	-	++	++	-	-	n.d.
	PaCa 5061	++	++	++	firm	+++	+++	-	-	++	+	-	-	n.d.
BZM-sensitive	HOS	-	-	-	loose	-	+	+	+	++	-	++	+++	+++
	MeWo	-	-	-	loose	-	+	+++	+	++	-	++	+++	+++

B

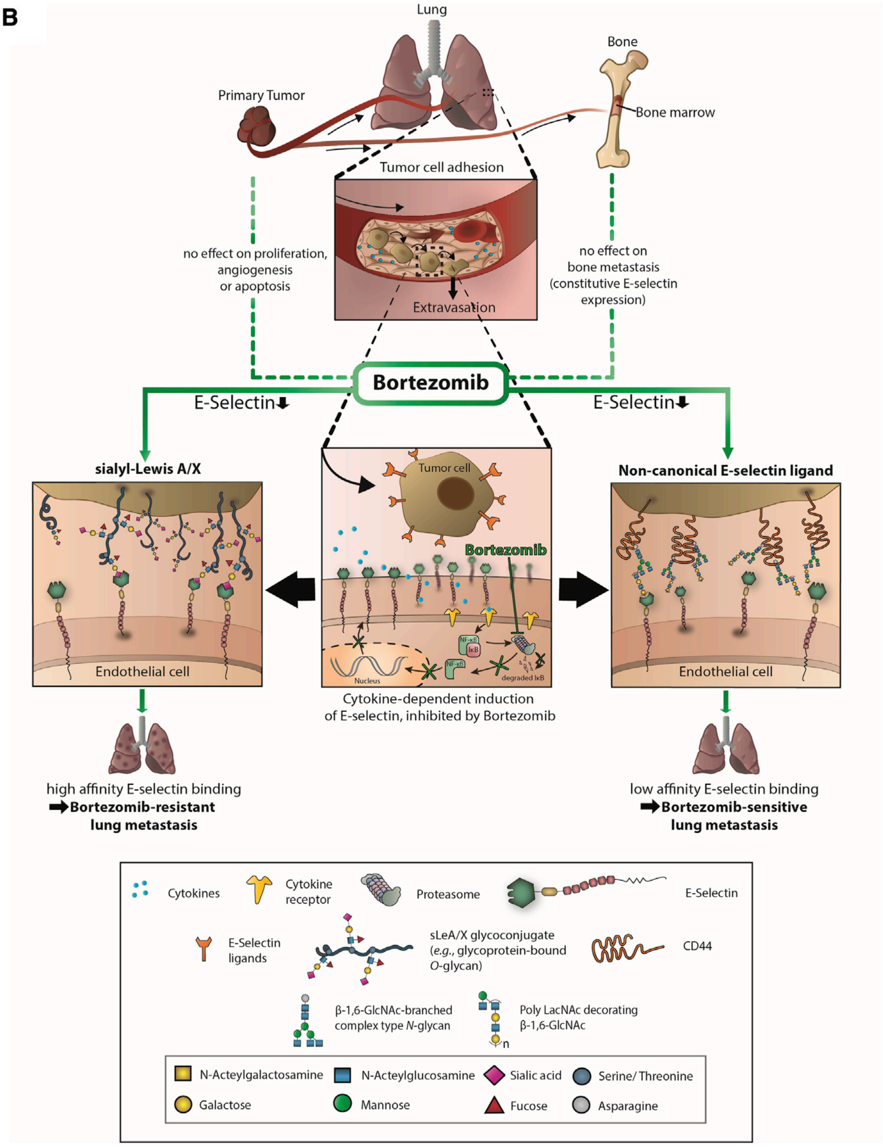

(legend on next page)

flow conditions only. This low affinity might explain why the incomplete effect of BZM on E-selectin expression is sufficient to block adhesion and thus metastasis specifically in this subset of tumor models. The observation that the non-canonical E-selectin ligands require flow conditions for functionality can be explained by the catch-bond effect, which means that lectin-sugar interactions are strengthened in the presence of (shear) force.[33] This shear-force-dependent catch-bond effect likely explains why sLeA/X-negative tumor cells, which do not bind to rhE-selectin under static conditions, do adhere under dynamic conditions.

Our proof-of-principle biochemical assays reveal that the adhesiveness of two BZM-sensitive tumor cell lines on ECs is reduced upon cleavage of terminal sialic acid (sialidase) after removing cell-surface glycoproteins (pronase) and after inhibiting *N*-glycosylation in the Golgi apparatus (Sw). The latter approach is accompanied by a reduced cell-surface presentation of β-1,6-GlcNAc branches and poly-LacNAc chains. Moreover, we demonstrate that these carbohydrates decorate CD44, a glycoprotein whose depletion strikingly abrogates endothelial adhesion and reduces pulmonary metastasis of the BZM-sensitive cells. We further show that CD44 is a substrate of MGAT5, the key enzyme of the biosynthesis of β-1,6-GlcNAc-branches, which are commonly elongated by poly-LacNAc.[29] Intriguingly, the same carbohydrate structure, i.e., sialylated poly-LacNAc, has already been described as a flow-dependent E-selectin ligand on human myeloid leukemia cells.[34] Therefore, sLeA/X-negative tumor cells appear to mimic properties of cells of the myeloid lineage for E-selectin binding. CD44 has been in the focus of cancer research for decades due to its multiple roles in tumor progression and invasion;[35–37] in particular, CD44 has been described as a mediator of adhesion of prostate and breast cancer cells to bone marrow endothelium[38] and as a major E-selectin ligand on breast[39,40] and colon cancer cells.[41] Here, we provide direct functional evidence for its pro-metastatic role using the most rigorous *in vivo* metastasis models available to date, i.e., spontaneous metastasis xenograft models.

In contrast, sLeA and/or sLeX are consistently present on tumor cells that exhibit the BZM-resistant phenotype. Such cells bind rhE-selectin under static conditions and adhere firmly on rhE-selectin under flow. The endothelial adhesions of these cells are much more stable than that of sLeA/X-negative tumor cells, suggesting a high-affinity E-selectin binding in the presence of sLeA/X. E-selectin binding, endothelial adhesion, and sLeA presentation on these cells mainly depend on *O*-GalNAc glycosylation. Correspondingly, these cells show a comparatively high expression of a core 2 *O*-glycan synthase (C2GNT2) and, after depletion of C2GNT2, sLeA expression and rhE-selectin binding are significantly reduced in our proof-of-principle experiment with one BZM-resistant cell line. Taken together, a considerable proportion of sLeA is apparently carried by core 2 *O*-glycans on these cells. The core 2 structure has been described as a common scaffold for sialylated Lewis structures,[42] and these have been shown to have enhanced E-selectin binding affinity when linked to core 2.[43] Therefore, the putative linkage of sLeA to core 2 could explain the high-affinity E-selectin binding of the sLeA-positive cells in the present study. Thus, C2GNT2 might be an attractive therapeutic target for tumors with BZM-resistant adhesion. However, this interpretation of the findings is complicated by the fact that the tested tumor cell lines with BZM-resistant adhesion do not respond to the pronase treatment. If sLeA was the key mediator of adhesion and carried by glycoprotein-bound *O*-glycans, pronase should have reduced sLeA, E-selectin binding, and endothelial adhesion of these tumor cells. Therefore, glycolipids should be considered as potential carriers of sLeA/high-affinity E-selectin ligands on tumor cells with BZM-resistant adhesion in future experiments. Moreover, it might well be that alterations in *O*-glycan maturation (upon treatment with GOB or depletion of C2GNT2) affect glycolipid synthesis and thereby sLeA expression, E-selectin binding, or endothelial adhesion.[8]

The role of sLeA as the determinant of the efficacy of BZM is further supported by our experiments using SKOV3 ovarian cancer cells. *In vitro*, SKOV3 cells lack sLeA/X and therefore show BZM-sensitive adhesion. *In vivo*, however, SKOV3 xenografts do express sLeA and therefore show BZM-resistant lung metastasis formation. The molecular basis for the sLeA induction *in vivo* remains to be explained but could simply be due to an influence of the 2D (*in vitro*) versus the 3D (*in vivo*) growth conditions on the glycosylation machinery of the tumor cells.[44,45] Similar observations have been made with gastric cancer spheroids by Balmana et al., who even demonstrated that the glycosylation patterns under 3D conditions more closely reflected the real patient situation.[46] Furthermore, we validated our cell-line-based observations by using PT models of human CRCs and observed the same relationship between sLeA/X expression, adhesiveness on rhE-selectin under flow, and BZM sensitivity, indicating that our findings have translational relevance.

One major issue of this study was to demonstrate the direct link between the anti-adhesive and anti-metastatic efficacy of BZM *in vitro* and *in vivo*, respectively. Of course, it is technically challenging to obtain such evidence since it would require real-time monitoring of spontaneous lung metastasis formation *in vivo*. To this end, we demonstrate the anti-adhesive efficacy of BZM on isolated murine lung ECs *in vitro* and within the pulmonary microcirculation *in situ*. Moreover, we exclude "off-target" effects of BZM at the PT site (no reduced tumor proliferation, angiogenesis, or increased apoptosis) and on metastatic outgrowth (no reduced number of cells per metastasis in the remaining lung metastases). Importantly, BZM

Figure 7. Summary and graphical abstract

(A) Characteristics of human tumor cells with BZM-resistant versus BZM-sensitive endothelial adhesion (from left to right): tumor-cell-surface sLeA/X expression and static rhE-selectin binding capacity, adhesion strength on rhE-selectin under flow, static adhesion strength on ECs, susceptibility of adhesions to E-selectin- or VCAM-1-blockade, adhesiveness on E-selectin-depleted ECs. Functional (pro-adhesive) role of α-2,3-sialic acid (cleaved by ND), *O*-glycans (reduced by GOB), *N*-glycans (reduced by SW), glycoproteins (cleaved by pronase [Pron]), and of CD44 (depleted by shRNA). n.d., not determined. (B) Graphical abstract.

has no effect on the metastatic burden in the bone marrow, which is known to constitutively express E-selectin independent of proteasomal activation.[17] Finally, the opposing findings with the SKOV3 model (*in vitro* versus *in vivo*) strongly support the presumed link as does the loss of anti-metastatic efficacy of BZM on CD44-depleted xenografts. In our view, these data provide convincing evidence that the anti-metastatic effect of BZM is due to its anti-adhesive activity. One remaining question is whether BZM affected cytokine levels in the circulation. If so, such an off-target effect would most likely have also led to reduced endothelial CAM expression.

Another issue was demonstrating that the anti-adhesive effect of BZM is due to the loss of E-selectin, particularly since the BZM-sensitive tumor cells not only depend on E-selectin but also on VCAM-1 for adhesion. Consistent with our hypothesis that the efficacy of BZM is determined by the E-selectin ligands on the tumor cells, we show that the remaining adhesions of BZM-sensitive cells on E-selectin-depleted ECs are not further reduced by BZM (although the EC treatment with BZM leads to reduced VCAM-1 expression). Ultimately, BZM loses its anti-metastatic efficacy when the xenograft experiment is conducted in E-selectin-knockout mice.

Detection of sLeA is feasible on cancer specimens (Figure S4C) so that patients could be stratified for the BZM treatment. While a negativity for sLeA can provide a first indication of the potential effectiveness of BZM at best, the inclusion of a positive marker indicating the presence of a particular low-affinity E-selectin ligand would certainly be more informative regarding patient stratification. Based on our data, this could be *N*-linked poly-LacNAc on CD44, the detection of which cannot yet be routinely collected. Such anti-metastatic BZM treatment could become relevant during periods associated with increased risk of CTC release such as surgery or biopsy[24–26] or for patients with a pathologic complete response of the PT but with detectable CTC counts in the neoadjuvant setting.[47] CTC counts also indicate worse prognosis in M1 patients,[48,49] so oligo-metastatic patients with detectable CTCs might benefit from the BZM-induced blockade of extravasation as well. BZM has been in clinical practice for more than 15 years and is considered to be well tolerated with manageable toxicity.[50]

Our findings (summarized in Figure 7) support the well-known, crucial role of the E-selectin-ligand interaction for distant metastasis formation and suggest BZM as a therapeutic option for solid cancers to reduce PT-induced E-selectin expression in the lung and thus lung metastasis. BZM has anti-adhesive, anti-metastatic potential on tumors lacking sLeA/X, which bind E-selectin through non-canonical, low-affinity, flow-dependent ligands. CD44 is one major determinant of adhesion and pulmonary metastasis of such tumor cells and binds E-selectin most likely through β-1,6-GlcNAc-branched *N*-glycans elongated with poly-LacNAc. Instead, inhibition of the core 2 *O*-glycan synthase C2GNT2 is useful to block sLeA synthesis in human tumors with BZM-resistant adhesion. SLeA/X mediate high-affinity, flow-independent E-selectin binding.

MATERIALS AND METHODS

For detailed information, please see the Supplemental information.

Spontaneous metastasis xenograft experiments

1×10^6 tumor cells (HT29, PaCa5061, HOS, HOS-shControl, HOS-shCD44, MeWo, MeWo-shControl, MeWo-shCD44, GC5023, SW2, or SKOV3) were s.c. injected below the right scapula of 12-week-old SCID (CB17/Icr-*Prkdc*^{scid}/IcrIcoCrl) or SCID *Sele*$^{-/-}$ mice (STOCK-Prkdc^{scid} Sele^{tm2Hyn}/Uke) in a volume of 200 μL as described.[12] For the treatment studies, mice received intraperitoneal injections of PBS (solvent control) or BZM (1.25 mg/kg body weight) starting on day 1 (d1) after tumor engraftment twice a week for the whole tumor growth period as illustrated in Figure 2. The general physical status of the animals was regularly monitored, and mice were sacrificed when the tumor reached maximal growth (up to 10% of the initial mouse body weight) or started to ulcerate. PTs and lungs were excised at necropsy for subsequent histology or DNA extraction. The femora and tibiae of mice from the HT29, PaCa5061, HOS, and MeWo xenograft models were flushed with 500 μL 0.9% NaCl solution to harvest the bone marrow for DNA extraction. The spontaneous metastatic cell loads in the lungs and bone marrow were quantified by *Alu*-PCR as described.[51] These *in vivo* experiments were approved by the local animal experiment approval committee (project nos. G19/21, G11/65, G15/19, G09/88).

Ex vivo lung perfusion model

For direct observations of the BZM-mediated effect on the dynamic adhesion of HOS cells to pulmonary microvessel endothelium, we used a murine *ex vivo* lung perfusion model as described[28] and as illustrated in Figure 2E. For a brief description, please see the Supplemental information. The *ex vivo* lung perfusion animal experiments were approved and assigned to the project no. G10/100.

Selectin/CAM expression on ECs, flow adhesion, and static E-selectin binding assays

Confluent monolayers of ECs were cultured in the presence or absence of 10 ng/μL rhTNFα or rhIL-1α (Peprotech) with or without pre-incubation with 10 μM BZM (Velcade, Janssen-Cilag) for 30 min (solvent control: PBS). After 4 h of cytokine stimulation, ECs were detached, and CAM expression was assessed by flow cytometry as described.[51] From additional EC samples, RNA was isolated, and cDNA was subjected to RT^2 qPCR profiler arrays for human ECM/CAM genes (Qiagen).

The adhesiveness of tumor cells on ECs (± rhTNFα or rhIL-1α, ± BZM) or rhE-selectin (rhE-selectin/immunoglobulin G [IgG]-Fc chimera, R&D Systems) was analyzed under physiological flow conditions in ibidiTreat μ-slide IV$^{0.4}$ flow chambers as described.[52] For adhesive events, we distinguished firm adhesion from rolling and tethering interactions (see illustration in Figure 1 and Richter et al.[52]). Endothelial proteins of interest were blocked using validated blocking monoclonal antibodies (mAbs) against E-selectin

(HAE-1f, BioLegend), ICAM-1 (BBIG-I1), and VCAM-1 (BBIG-V1) (both from R&D Systems) at concentrations indicated by the manufacturer, which were given to cytokine-stimulated ECs 30 min prior to the flow adhesion assay. The stability of endothelial adhesions was challenged by raising the laminar flow rate to a maximum of 200 mL/h. The static rhE-selectin binding capacity of native and pre-treated tumor cells was assessed by flow cytometry as previously described.[14]

Determination of pro-adhesive carbohydrate structures at the tumor cell surface and their alteration by enzymatic, chemical, and pharmacologic treatments

The canonical E-selectin ligands were analyzed at the tumor cell surface by flow cytometry using mAbs against sLeX (CSLEX1) or sLeA (121SLE) as described.[51] β-1,6-GlcNAc branches and poly-LacNAc were determined using biotinylated PHA-L and DSL, respectively (Vector Labs). As "isotype" controls, lectins were applied after sugar inhibition with bovine thyroglobulin (Sigma) and chitin hydrolysate (Vector Labs), respectively. Lectins were labeled with streptavidin-APC (Sigma) for flow cytometry.

To test their functional importance for endothelial adhesion and E-selectin binding, sialic-acid-containing sugar residues were enzymatically cleaved using 10 mU/mL ND (from *Clostridium perfringens*, Roche) added to the tumor cell culture at 37°C for 1h under serum-free conditions. Likewise, glycoproteins were cleaved by 1 mg/mL pronase (from *Streptomyces griseus*) added to the tumor cell culture at 37°C for 45 min under serum-free conditions. *O*-GalNAc-glycosylation was chemically inhibited by adding 2 mM GOB (Sigma) for 72 h to the culture medium (solvent control: cell culture medium). *N*-glycosylation was inhibited using 2 μM synthetic Sw (Sigma) for 72 h (solvent control: methanol). Detrimental effects of all treatments on tumor cell viability were excluded by propidium iodide uptake analyses (flow cytometry [FC]).

SUPPLEMENTAL INFORMATION

Supplemental information can be found online at https://doi.org/10.1016/j.ymthe.2022.01.017.

ACKNOWLEDGMENTS

This work was funded by a joint German Research Foundation grant to T.L. and R.K. (project ID 325043972) and a Roggenbuck Foundation grant to T.L. H.M.D. is funded by the Veterans Administration Merit Award, and A.S. is funded by the German Research Foundation (SCHW 407/17-1). The authors thank T. Gosau, M. Märker, and J. Schröder-Schwarz for their excellent technical assistance and Professor J. Dietl (Department of Obstetrics and Gynecology, Würzburg University Hospital) for providing the ovarian cancer specimens. The authors are grateful for financial support from the German Cancer Aid through the University Cancer Center Hamburg.

AUTHOR CONTRIBUTIONS

Conceptualization, T.L., U.V., D.W., and U.S.; methodology, T.L., U.V., D.W., A.-K.A., C.P., A.T.B., A.S., K.R., R.K., H.M.D., A.B., and U.S.; investigation, T.L., U.V., D.W., H.M., V.L., A.-K.A., S.S., S.G., L.S., C.P., A.-M.D., C.S., A.B., A.T.B., E.B., C.B., and V.A.; writing – original draft, T.L. and E.B.; writing – review & editing, U.V., D.W., V.L., A.B., A.S., K.P., and U.S.; funding acquisition, T.L. and R.K.; resources, K.R., T.G., J.K.H., and A.B.; supervision, T.L., A.S., R.K., H.M.D., J.K.H., A.B., K.P., and U.S. All authors read and approved the final manuscript.

DECLARATION OF INTERESTS

The authors declare no competing interests.

REFERENCES

1. Lambert, A.W., Pattabiraman, D.R., and Weinberg, R.A. (2017). Emerging biological principles of metastasis. Cell *168*, 670–691. https://doi.org/10.1016/j.cell.2016.11.037.
2. Joosse, S.A., Gorges, T.M., and Pantel, K. (2015). Biology, detection, and clinical implications of circulating tumor cells. EMBO Mol. Med. *7*, 1–11. https://doi.org/10.15252/emmm.201303698.
3. Strilic, B., and Offermanns, S. (2017). Intravascular survival and extravasation of tumor cells. Cancer Cell *32*, 282–293. https://doi.org/10.1016/j.ccell.2017.07.001.
4. Dasgupta, A., Lim, A.R., and Ghajar, C.M. (2017). Circulating and disseminated tumor cells: harbingers or initiators of metastasis? Mol. Oncol. *11*, 40–61. https://doi.org/10.1002/1878-0261.12022.
5. Reymond, N., d'Agua, B.B., and Ridley, A.J. (2013). Crossing the endothelial barrier during metastasis. Nat. Rev. Cancer *13*, 858–870. https://doi.org/10.1038/nrc3628.
6. Strell, C., and Entschladen, F. (2008). Extravasation of leukocytes in comparison to tumor cells. Cell Commun. Signal. *6*, 10.
7. St Hill, C.A. (2011). Interactions between endothelial selectins and cancer cells regulate metastasis. Front. Biosci. (Landmark Ed.) *16*, 3233–3251.
8. Starzonek, S., Maar, H., Labitzky, V., Wicklein, D., Rossdam, C., Buettner, F.F.R., Wolters-Eisfeld, G., Guengoer, C., Wagener, C., Schumacher, U., and Lange, T. (2020). Systematic analysis of the human tumor cell binding to human vs. murine E- and P-selectin under static vs. dynamic conditions. Glycobiology *30*, 695–709. https://doi.org/10.1093/glycob/cwaa019.
9. Shirure, V.S., Henson, K.A., Schnaar, R.L., Nimrichter, L., and Burdick, M.M. (2011). Gangliosides expressed on breast cancer cells are E-selectin ligands. Biochem. Biophys. Res. Commun. *406*, 423–429. https://doi.org/10.1016/j.bbrc.2011.02.061.
10. Liang, S., and Dong, C. (2008). Integrin VLA-4 enhances sialyl-Lewisx/a-negative melanoma adhesion to and extravasation through the endothelium under low flow conditions. Am. J. Physiol. Cell Physiol. *295*, C701–C707. https://doi.org/10.1152/ajpcell.00245.2008.
11. Gebauer, F., Wicklein, D., Stubke, K., Nehmann, N., Schmidt, A., Salamon, J., Peldschus, K., Nentwich, M.F., Adam, G., Tolstonog, G., et al. (2013). Selectin binding is essential for peritoneal carcinomatosis in a xenograft model of human pancreatic adenocarcinoma in pfp−/rag2− mice. Gut *62*, 741–750.
12. Köhler, S., Ullrich, S., Richter, U., and Schumacher, U. (2010). E-/P-selectins and colon carcinoma metastasis: first in vivo evidence for their crucial role in a clinically relevant model of spontaneous metastasis formation in the lung. Br. J. Cancer *102*, 602–609.
13. Heidemann, F., Schildt, A., Schmid, K., Bruns, O.T., Riecken, K., Jung, C., Ittrich, H., Wicklein, D., Reimer, R., Fehse, B., et al. (2014). Selectins mediate small cell lung cancer systemic metastasis. PLoS One *9*, e92327. https://doi.org/10.1371/journal.pone.0092327.
14. Wicklein, D., Schmidt, A., Labitzky, V., Ullrich, S., Valent, P., and Schumacher, U. (2013). E- and p-selectins are essential for repopulation of chronic myelogenous and chronic eosinophilic leukemias in a scid mouse xenograft model. PLoS One *8*, e70139.
15. Stübke, K., Wicklein, D., Herich, L., Schumacher, U., and Nehmann, N. (2012). Selectin-deficiency reduces the number of spontaneous metastases in a xenograft model of human breast cancer. Cancer Lett. *321*, 89–99.

16. Haraldsen, G., Kvale, D., Lien, B., Farstad, I.N., and Brandtzaeg, P. (1996). Cytokine-regulated expression of E-selectin, intercellular adhesion molecule-1 (ICAM-1), and vascular cell adhesion molecule-1 (VCAM-1) in human microvascular endothelial cells. J. Immunol. *156*, 2558–2565.
17. Schweitzer, K.M., Drager, A.M., van der Valk, P., Thijsen, S.F., Zevenbergen, A., Theijsmeijer, A.P., van der Schoot, C.E., and Langenhuijsen, M.M. (1996). Constitutive expression of E-selectin and vascular cell adhesion molecule-1 on endothelial cells of hematopoietic tissues. Am. J. Pathol. *148*, 165–175.
18. McAllister, S.S., and Weinberg, R.A. (2014). The tumour-induced systemic environment as a critical regulator of cancer progression and metastasis. Nat. Cell Biol. *16*, 717–727. https://doi.org/10.1038/ncb3015.
19. Hiratsuka, S., Goel, S., Kamoun, W.S., Maru, Y., Fukumura, D., Duda, D.G., and Jain, R.K. (2011). Endothelial focal adhesion kinase mediates cancer cell homing to discrete regions of the lungs via E-selectin up-regulation. Proc. Natl. Acad. Sci. U S A *108*, 3725–3730. https://doi.org/10.1073/pnas.1100446108.
20. Egberts, J.H., Cloosters, V., Noack, A., Schniewind, B., Thon, L., Klose, S., Kettler, B., von Forstner, C., Kneitz, C., Tepel, J., et al. (2008). Anti-tumor necrosis factor therapy inhibits pancreatic tumor growth and metastasis. Cancer Res. *68*, 1443–1450. https://doi.org/10.1158/0008-5472.CAN-07-5704.
21. Michalaki, V., Syrigos, K., Charles, P., and Waxman, J. (2004). Serum levels of IL-6 and TNF-alpha correlate with clinicopathological features and patient survival in patients with prostate cancer. Br. J. Cancer *90*, 2312–2316. https://doi.org/10.1038/sj.bjc.6601814.
22. Gilmore, T.D. (2006). Introduction to NF-kappaB: players, pathways, perspectives. Oncogene *25*, 6680–6684. https://doi.org/10.1038/sj.onc.1209954.
23. Sunwoo, J.B., Chen, Z., Dong, G., Yeh, N., Crowl Bancroft, C., Sausville, E., Adams, J., Elliott, P., and Van Waes, C. (2001). Novel proteasome inhibitor PS-341 inhibits activation of nuclear factor-kappa B, cell survival, tumor growth, and angiogenesis in squamous cell carcinoma. Clin. Cancer Res. *7*, 1419–1428.
24. Bosch, B., Guller, U., Schnider, A., Maurer, R., Harder, F., Metzger, U., and Marti, W.R. (2003). Perioperative detection of disseminated tumour cells is an independent prognostic factor in patients with colorectal cancer. Br. J. Surg. *90*, 882–888. https://doi.org/10.1002/bjs.4129.
25. Mathenge, E.G., Dean, C.A., Clements, D., Vaghar-Kashani, A., Photopoulos, S., Coyle, K.M., Giacomantonio, M., Malueth, B., Nunokawa, A., Jordan, J., et al. (2014). Core needle biopsy of breast cancer tumors increases distant metastases in a mouse model. Neoplasia *16*, 950–960. https://doi.org/10.1016/j.neo.2014.09.004.
26. Weitz, J., Kienle, P., Lacroix, J., Willeke, F., Benner, A., Lehnert, T., Herfarth, C., and von Knebel Doeberitz, M. (1998). Dissemination of tumor cells in patients undergoing surgery for colorectal cancer. Clin. Cancer Res. *4*, 343–348.
27. Fehrenbach, M.L., Cao, G., Williams, J.T., Finklestein, J.M., and Delisser, H.M. (2009). Isolation of murine lung endothelial cells. Am. J. Physiol. Lung Cell Mol. Physiol. *296*, L1096–L1103. https://doi.org/10.1152/ajplung.90613.2008.
28. Kiefmann, R., Rifkind, J.M., Nagababu, E., and Bhattacharya, J. (2008). Red blood cells induce hypoxic lung inflammation. Blood *111*, 5205–5214. https://doi.org/10.1182/blood-2007-09-113902.
29. Cummings, R.D., and Liu, F.T. (2009). Galectins. In Essentials of Glycobiology, 2nd Edition, A. Varki, R.D. Cummings, J.D. Esko, H.H. Freeze, P. Stanley, C.R. Bertozzi, G.W. Hart, and M.E. Etzler, eds. (Cold Spring Harbor (NY)).
30. Alvarez-Manilla, G., Troupe, K., Fleming, M., Martinez-Uribe, E., and Pierce, M. (2010). Comparison of the substrate specificities and catalytic properties of the sister N-acetylglucosaminyltransferases, GnT-V and GnT-Vb (IX). Glycobiology *20*, 166–174. https://doi.org/10.1093/glycob/cwp158.
31. Cummings, R.D., and Kornfeld, S. (1984). The distribution of repeating [Gal beta 1,4GlcNAc beta 1,3] sequences in asparagine-linked oligosaccharides of the mouse lymphoma cell lines BW5147 and PHAR 2.1. J. Biol. Chem. *259*, 6253–6260.
32. Forones, N.M., and Tanaka, M. (1999). CEA and CA 19-9 as prognostic indexes in colorectal cancer. Hepatogastroenterology *46*, 905–908.
33. Thomas, W. (2008). Catch bonds in adhesion. Annu. Rev. Biomed. Eng. *10*, 39–57. https://doi.org/10.1146/annurev.bioeng.10.061807.160427.
34. Handa, K., Stroud, M.R., and Hakomori, S. (1997). Sialosyl-fucosyl Poly-LacNAc without the sialosyl-Lex epitope as the physiological myeloid cell ligand in E-selectin-dependent adhesion: studies under static and dynamic flow conditions. Biochemistry *36*, 12412–12420. https://doi.org/10.1021/bi971181t.
35. Chen, C., Zhao, S., Karnad, A., and Freeman, J.W. (2018). The biology and role of CD44 in cancer progression: therapeutic implications. J. Hematol. Oncol. *11*, 64. https://doi.org/10.1186/s13045-018-0605-5.
36. McFarlane, S., Coulter, J.A., Tibbits, P., O'Grady, A., McFarlane, C., Montgomery, N., Hill, A., McCarthy, H.O., Young, L.S., Kay, E.W., et al. (2015). CD44 increases the efficiency of distant metastasis of breast cancer. Oncotarget *6*, 11465–11476. https://doi.org/10.18632/oncotarget.3410.
37. Senbanjo, L.T., and Chellaiah, M.A. (2017). CD44: a multifunctional cell surface adhesion receptor is a regulator of progression and metastasis of cancer cells. Front. Cell Dev. Biol. *5*, 18. https://doi.org/10.3389/fcell.2017.00018.
38. Draffin, J.E., McFarlane, S., Hill, A., Johnston, P.G., and Waugh, D.J. (2004). CD44 potentiates the adherence of metastatic prostate and breast cancer cells to bone marrow endothelial cells. Cancer Res. *64*, 5702–5711. https://doi.org/10.1158/0008-5472.CAN-04-0389.
39. Zen, K., Liu, D.Q., Guo, Y.L., Wang, C., Shan, J., Fang, M., Zhang, C.Y., and Liu, Y. (2008). CD44v4 is a major E-selectin ligand that mediates breast cancer cell transendothelial migration. PLoS One *3*, e1826. https://doi.org/10.1371/journal.pone.0001826.
40. Shirure, V.S., Liu, T., Delgadillo, L.F., Cuckler, C.M., Tees, D.F., Benencia, F., Goetz, D.J., and Burdick, M.M. (2015). CD44 variant isoforms expressed by breast cancer cells are functional E-selectin ligands under flow conditions. Am. J. Physiol. Cell Physiol. *308*, C68–C78. https://doi.org/10.1152/ajpcell.00094.2014.
41. Hanley, W.D., Burdick, M.M., Konstantopoulos, K., and Sackstein, R. (2005). CD44 on LS174T colon carcinoma cells possesses E-selectin ligand activity. Cancer Res. *65*, 5812–5817. https://doi.org/10.1158/0008-5472.CAN-04-4557.
42. Brockhausen, I., Schachter, H., and Stanley, P. (2009). O-GalNAc glycans. In Essentials of Glycobiology, 2nd edition Edition, A. Varki, R.D. Cummings, J.D. Esko, H.H. Freeze, P. Stanley, C.R. Bertozzi, G.W. Hart, and M.E. Etzler, eds. (Cold Spring Harbor Laboratory Press).
43. St Hill, C.A., Bullard, K.M., and Walcheck, B. (2005). Expression of the high-affinity selectin glycan ligand C2-O-sLeX by colon carcinoma cells. Cancer Lett. *217*, 105–113. https://doi.org/10.1016/j.canlet.2004.06.038.
44. Geng, Y., Chandrasekaran, S., Hsu, J.W., Gidwani, M., Hughes, A.D., and King, M.R. (2013). Phenotypic switch in blood: effects of pro-inflammatory cytokines on breast cancer cell aggregation and adhesion. PLoS One *8*, e54959. https://doi.org/10.1371/journal.pone.0054959.
45. Schumacher, U., Adam, E., Flavell, D.J., Boehm, D., Brooks, S.A., and Leathem, A.J. (1994). Glycosylation patterns of the human colon cancer cell line HT-29 detected by Helix pomatia agglutinin and other lectins in culture, in primary tumours and in metastases in SCID mice. Clin. Exp. Metastasis *12*, 398–404. https://doi.org/10.1007/bf01755883.
46. Balmana, M., Mereiter, S., Diniz, F., Feijao, T., Barrias, C.C., and Reis, C.A. (2018). Multicellular human gastric-cancer spheroids mimic the glycosylation phenotype of gastric carcinomas. Molecules *23*, 2815. https://doi.org/10.3390/molecules23112815.
47. Riethdorf, S., Muller, V., Loibl, S., Nekljudova, V., Weber, K., Huober, J., Fehm, T., Schrader, I., Hilfrich, J., Holms, F., et al. (2017). Prognostic impact of circulating tumor cells for breast cancer patients treated in the neoadjuvant "Geparquattro" trial. Clin. Cancer Res. *23*, 5384–5393. https://doi.org/10.1158/1078-0432.CCR-17-0255.
48. Cristofanilli, M., Budd, G.T., Ellis, M.J., Stopeck, A., Matera, J., Miller, M.C., Reuben, J.M., Doyle, G.V., Allard, W.J., Terstappen, L.W., and Hayes, D.F. (2004). Circulating tumor cells, disease progression, and survival in metastatic breast cancer. N. Engl. J. Med. *351*, 781–791. https://doi.org/10.1056/NEJMoa040766.
49. Keller, L., and Pantel, K. (2019). Unravelling tumour heterogeneity by single-cell profiling of circulating tumour cells. Nat. Rev. Cancer *19*, 553–567. https://doi.org/10.1038/s41568-019-0180-2.

50. Mohan, M., Matin, A., and Davies, F.E. (2017). Update on the optimal use of bortezomib in the treatment of multiple myeloma. Cancer Manag. Res. *9*, 51–63. https://doi.org/10.2147/CMAR.S105163.

51. Lange, T., Kupfernagel, M., Wicklein, D., Gebauer, F., Maar, H., Brugge, K., Müller, I., Simon, R., Schlomm, T., Sauter, G., and Schumacher, U. (2014). Aberrant presentation of HPA-reactive carbohydrates implies Selectin-independent metastasis formation in human prostate cancer. Clin. Cancer Res. *20*, 1791–1802. https://doi.org/10.1158/1078-0432.CCR-13-2308.

52. Richter, U., Schroder, C., Wicklein, D., Lange, T., Geleff, S., Dippel, V., Schumacher, U., and Klutmann, S. (2011). Adhesion of small cell lung cancer cells to E- and P-selectin under physiological flow conditions: implications for metastasis formation. Histochem. Cell Biol. *135*, 499–512. https://doi.org/10.1007/s00418-011-0804-4.

YMTHE, Volume *30*

Supplemental Information

Tumor cell E-selectin ligands determine partial efficacy of bortezomib on spontaneous lung metastasis formation of solid human tumors *in vivo*

Tobias Lange, Ursula Valentiner, Daniel Wicklein, Hanna Maar, Vera Labitzky, Ann-Kristin Ahlers, Sarah Starzonek, Sandra Genduso, Lisa Staffeldt, Carolin Pahlow, Anna-Maria Dück, Christine Stürken, Anke Baranowsky, Alexander T. Bauer, Etmar Bulk, Albrecht Schwab, Kristoffer Riecken, Christian Börnchen, Rainer Kiefmann, Valsamma Abraham, Horace M. DeLisser, Timo Gemoll, Jens K. Habermann, Andreas Block, Klaus Pantel, and Udo Schumacher

Supplemental Figures and Legends

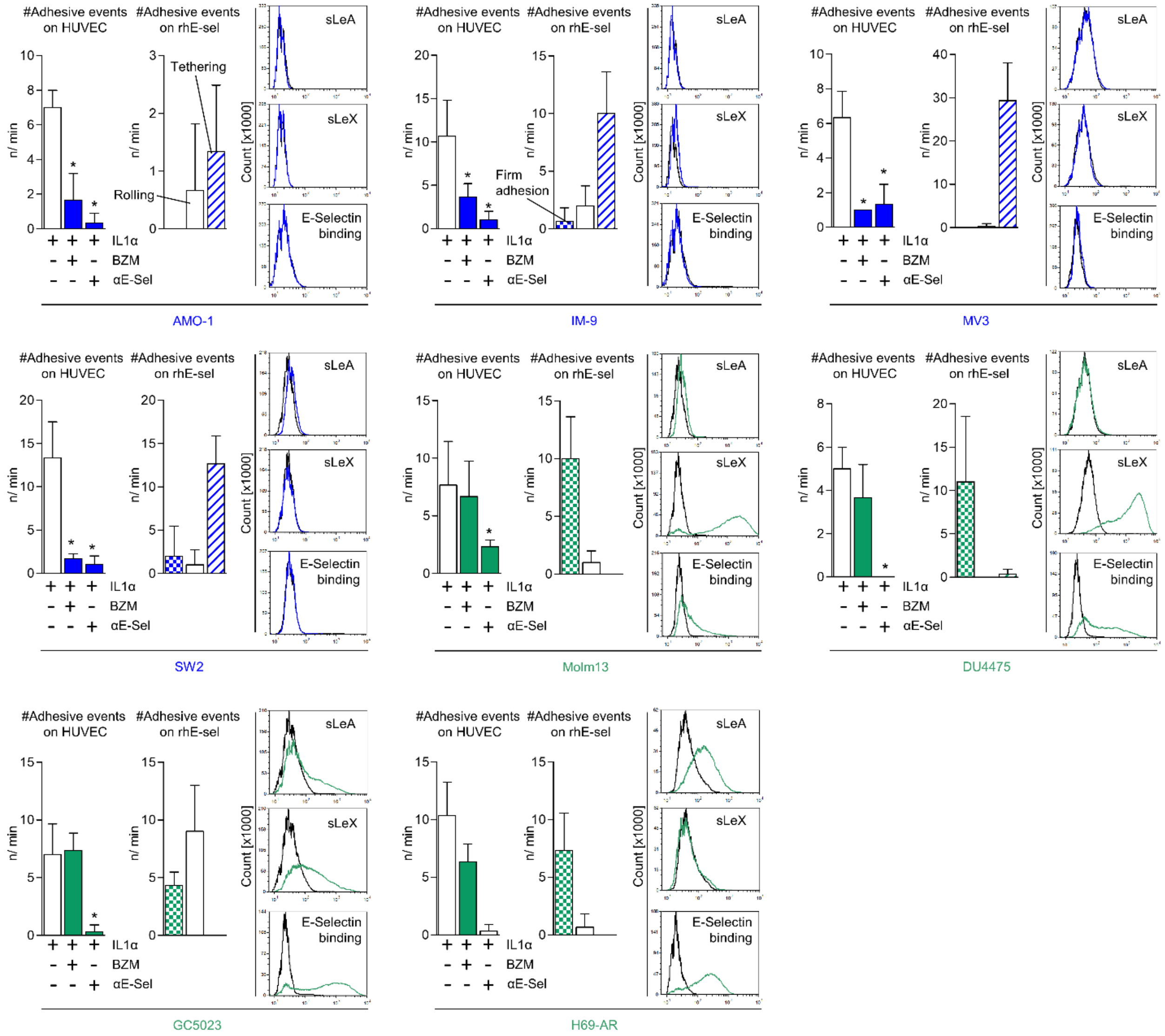

Figure S1: Verification of the relationship between adhesive properties of the tumor cells and anti-adhesive efficacy of BZM by further human tumor cell lines. Numbers of adhesive events of indicated tumor cells under flow conditions on ECs treated +/-IL-1α +/-BZM +/-E-selectin-blocking antibody as well as on immobilized rhE-selectin. Representative histograms of tumor cell surface sLeA/X expression and static E-selectin binding capacity. Bar charts represent mean ± SD of three replicates; black lines in histograms represent isotype controls (sLeA, sLeX) or binding of IgG1-Fc (rhE-selectin binding); **p*<0.05.

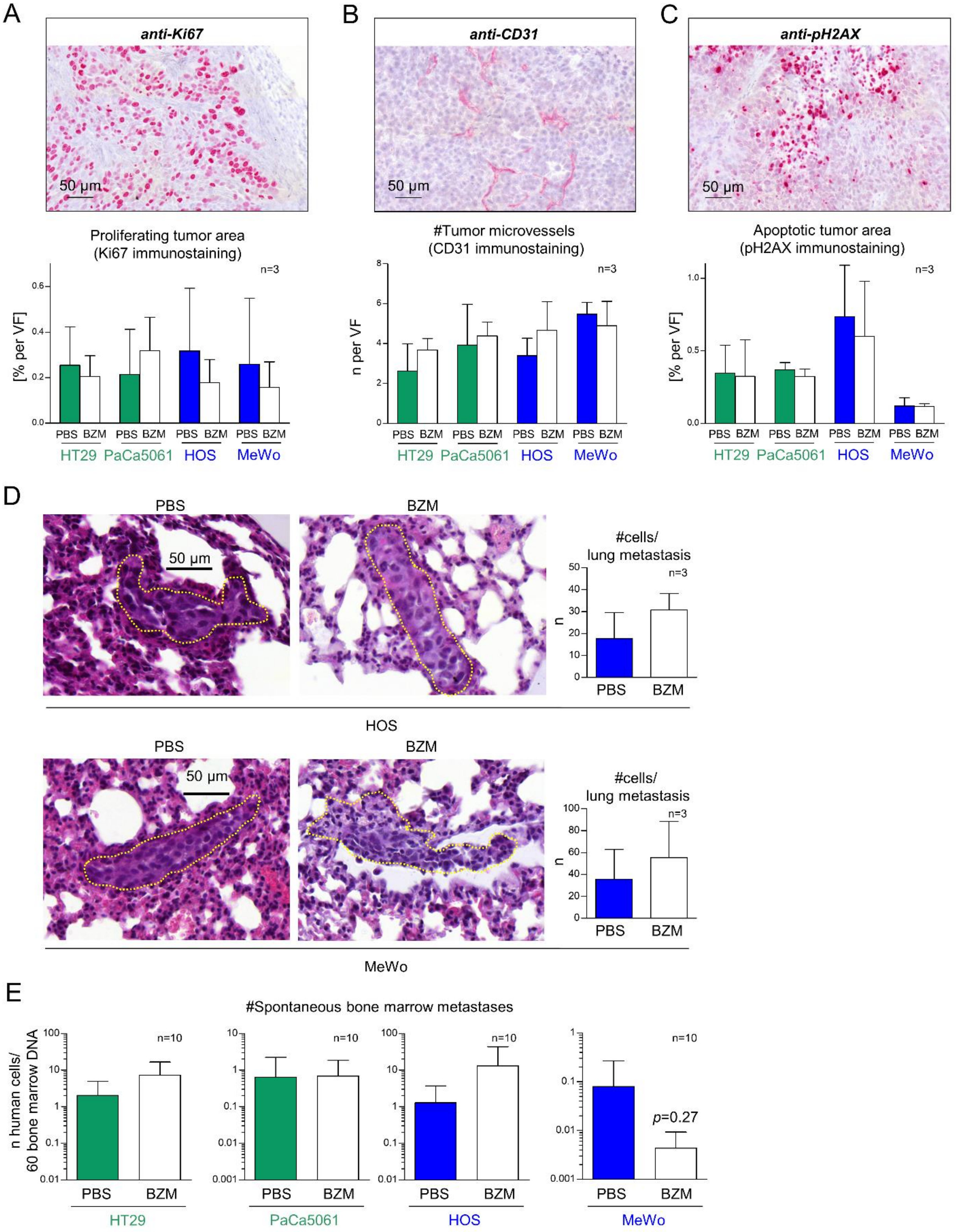
A
anti-Ki67
50 µm
Proliferating tumor area
(Ki67 immunostaining)
n=3
[% per VF]
PBS BZM PBS BZM PBS BZM PBS BZM
HT29 PaCa5061 HOS MeWo
B
anti-CD31
50 µm
#Tumor microvessels
(CD31 immunostaining)
n=3
n per VF
C
anti-pH2AX
50 µm
Apoptotic tumor area
(pH2AX immunostaining)
n=3
[% per VF]
D
PBS
BZM
50 µm
#cells/
lung metastasis
n=3
n
HOS
MeWo
E
#Spontaneous bone marrow metastases
n human cells/
60 bone marrow DNA
n=10
p=0.27
PBS BZM
HT29
PaCa5061
HOS
MeWo

Figure S2: Exclusion of potential 'off-target' effects of BZM. Immunostaining-based quantification of Ki67-positive xenograft primary tumor cells (A), CD31-positive xenograft primary tumor microvessels (B) and p2H2AX-positive xenograft primar tumor cells in the indicated xenograft models and treatment groups (C). Number of cells per lung metastasis in PBS vs. BZM-treated mice carrying the annotated s.c. xenografts (D). Number of spontaneous bone marrow metastases in PBS vs. BZM-treated mice bearing the indicated s.c. xenografts (E). Bone marrow endothelium is known to constitutively express E-selectin [17]. Bar charts in (A-C) represent mean ± SD from n=3, each containing up to 27 viewing fields per tumor. Bar charts in (D) represent mean ± SD of n=3. Bar charts in (E) represent mean ± SD of n=10.

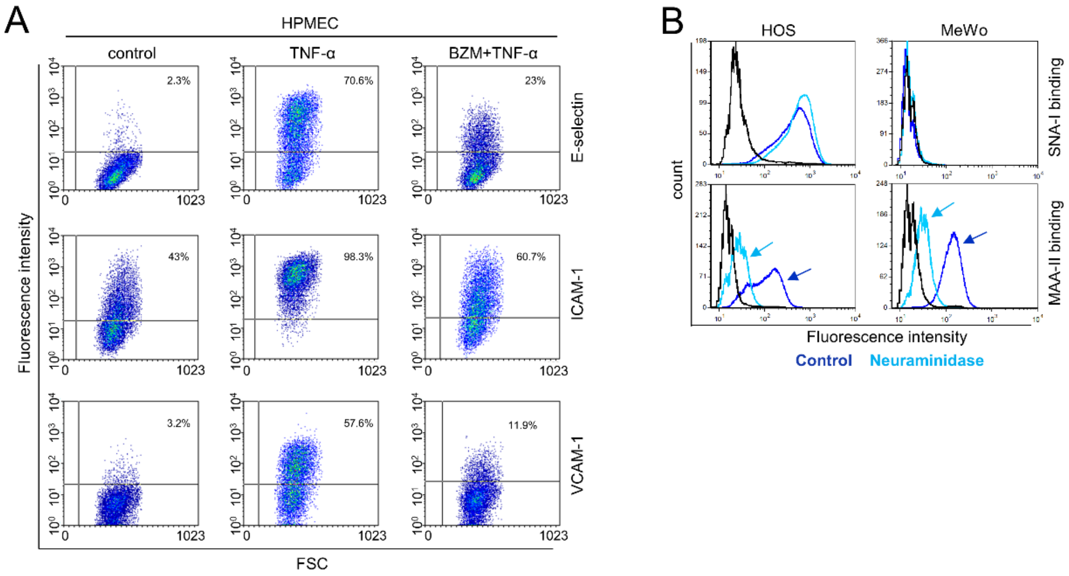

Figure S3: BZM effects on cytokine-mediated induction of CAMs in HPMEC and detection of α-2,3- and α-2,6-sialic acid on sLeA/X-negative tumor cells. Flow cytometric analyses of E-selectin, ICAM-1 and VCAM-1 after TNF-α stimulation and pre-incubation with BZM in HPMEC (A). MAA-II and SNA-I lectin binding towards HOS and MeWo cells before (dark blue) and after (light blue) neuraminidase treatment as indicated (B).

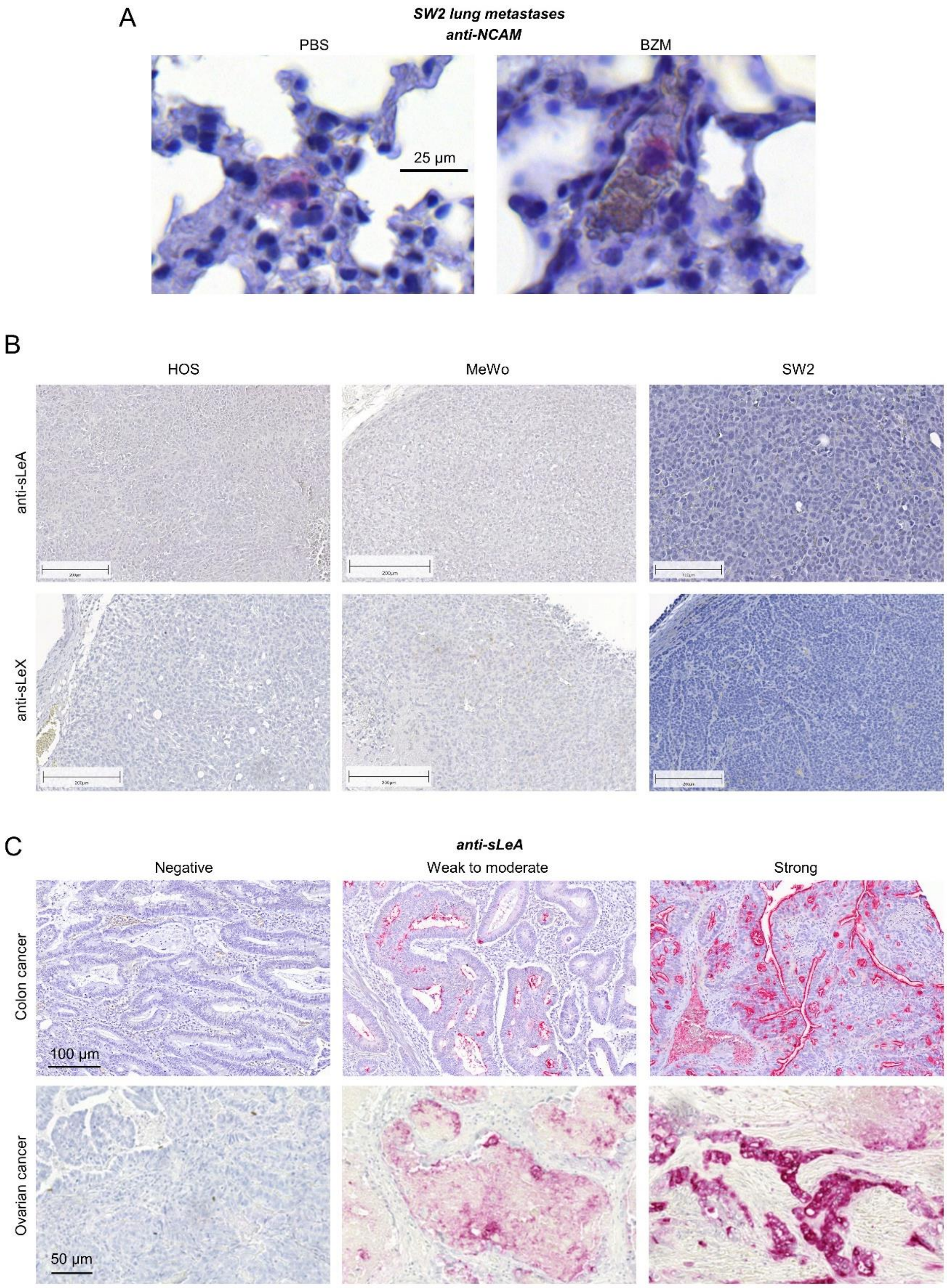

Figure S4: Detection of SW2 single cell metastases and determination of sLeA. Detection of human small cell lung cancer SW2 cells in the lungs of mice bearing s.c. xenograft tumors. In both

groups (PBS vs. BZM), only single cells could be found in the lungs (visualized by anti-NCAM immunostaining) (A). Other than SKOV3 (Fig. 6D), HOS, MeWo and SW2 xenografts fail to upregulate sLeA or sLeX *in vivo* (B). Detection of differential sLeA levels in primary patient material of colon and ovarian cancer by IHC (B).

Table S1: qPCR profiler array human glycosylation/ glucosidase genes, see Fig. 4E.

	2^-ΔCt			Fold Regulation	
Gene symbol	HT29	HOS	MeWo	HOS *vs.* HT29	MeWo *vs.* HT29
A4GNT	2.2E-04	5.4E-05	5.8E-04	-4.04	2.65
AGA	1.5E-02	3.7E-03	2.0E-02	-3.95	1.34
B3GALTL	2.5E-02	9.1E-03	2.8E-02	-2.68	1.13
B3GNT2	8.1E-02	2.5E-02	7.5E-02	-3.28	-1.08
B3GNT3	4.3E-02	1.7E-03	2.4E-03	-24.84	-17.9
B3GNT4	7.1E-04	6.3E-04	1.1E-03	-1.11	1.55
B3GNT8	5.7E-04	2.7E-04	1.5E-03	-2.09	2.58
B4GALT1	1.6E-01	3.2E-02	1.0E-01	-5.08	-1.6
B4GALT2	6.5E-02	4.2E-02	1.1E-01	-1.56	1.62
B4GALT3	5.8E-02	2.6E-02	6.1E-02	-2.19	1.05
B4GALT5	7.8E-02	5.3E-02	1.5E-01	-1.47	1.91
C1GALT1	1.2E-01	4.5E-02	9.7E-02	-2.66	-1.24
C1GALT1C1	5.2E-02	4.1E-02	4.9E-02	-1.27	-1.05
EDEM1	2.3E-02	5.7E-02	1.1E-01	2.45	4.91
EDEM2	2.5E-02	1.9E-02	3.4E-02	-1.29	1.39
EDEM3	4.7E-02	3.4E-02	3.4E-02	-1.38	-1.38
FUCA1	5.3E-02	3.9E-03	1.6E-02	-13.68	-3.27
FUCA2	9.8E-02	9.5E-02	2.9E-01	-1.02	2.98
FUT11	2.4E-02	2.1E-02	7.9E-02	-1.12	3.26
FUT8	4.5E-03	1.6E-01	3.9E-02	34.89	8.67
GALNT1	2.3E-02	8.2E-02	1.6E-01	3.56	6.9
GALNT10	7.8E-03	4.8E-02	7.9E-03	6.12	1.01
GALNT11	1.5E-02	5.9E-03	5.4E-02	-2.59	3.52
GALNT12	1.1E-01	2.6E-03	7.7E-03	-41.49	-14.24
GALNT13	5.7E-05	2.9E-05	4.1E-05	-1.99	-1.38
GALNT14	5.5E-02	1.6E-02	3.7E-05	-3.42	-1491.45
GALNT2	2.4E-02	3.7E-02	2.2E-01	1.51	9.11
GALNT3	1.8E-01	2.1E-05	1.4E-01	-8569.52	-1.26
GALNT4	1.5E-02	6.0E-03	6.9E-03	-2.52	-2.19
GALNT6	1.4E-03	1.1E-02	4.0E-04	7.33	-3.56
GALNT7	4.6E-02	3.3E-02	2.2E-02	-1.38	-2.05
GALNT8	2.0E-04	1.6E-05	3.7E-05	-12.25	-5.43
GALNT9	1.8E-04	1.4E-04	5.6E-04	-1.31	3.15
GALNTL1	1.1E-04	3.7E-03	1.3E-04	34.41	1.15
GALNTL5	4.9E-05	1.9E-05	8.3E-05	-2.54	1.7
GALNTL6	2.1E-05	1.6E-05	6.9E-05	-1.3	3.22
GANAB	5.0E-01	4.3E-01	6.5E-01	-1.16	1.29
GCNT1	1.2E-03	1.5E-03	3.4E-03	1.21	2.72
C2GNT2	9.2E-01	1.7E-04	1.1E-03	-5499.17	-856.61
GCNT4	2.9E-04	6.3E-04	3.9E-04	2.16	1.32
GLB1	6.8E-02	5.2E-02	5.5E-02	-1.3	-1.24

GNPTAB	2.3E-02	1.2E-02	2.4E-01	-1.87	10.46
GNPTG	3.3E-02	1.7E-02	8.8E-02	-1.91	2.68
HEXA	7.7E-03	1.1E-02	1.3E-01	1.46	16.53
HEXB	1.3E-01	6.5E-02	3.1E-01	-1.91	2.47
MAN1A1	5.9E-02	4.1E-02	6.8E-04	-1.45	-86.97
MAN1A2	3.3E-02	1.1E-02	4.6E-02	-3.06	1.42
MAN1B1	6.2E-02	2.9E-02	8.4E-02	-2.15	1.35
MAN1C1	1.6E-04	2.3E-03	7.4E-04	14.57	4.68
MAN2A1	4.2E-02	1.9E-02	3.6E-02	-2.19	-1.16
MAN2A2	2.4E-02	4.3E-03	4.6E-02	-5.59	1.91
MAN2B1	1.6E-02	2.1E-02	1.7E-02	1.3	1.09
MANBA	1.9E-02	5.0E-03	2.3E-02	-3.71	1.24
MGAT1	2.8E-02	1.5E-02	7.9E-02	-1.83	2.86
MGAT2	6.8E-03	2.2E-02	1.6E-02	3.17	2.34
MGAT3	3.7E-03	1.2E-04	3.7E-05	-29.54	-98.53
MGAT4A	3.6E-03	1.5E-04	1.3E-02	-23.34	3.54
MGAT4B	1.8E-01	5.7E-02	1.1E-01	-3.08	-1.66
MGAT4C	2.1E-05	1.6E-05	3.7E-05	-1.3	1.72
MGAT5	9.0E-02	2.2E-02	7.4E-02	-4.09	-1.21
MGAT5B	1.6E-04	1.0E-02	1.0E-01	63.77	638.03
MOGS	4.6E-02	3.0E-02	6.3E-02	-1.54	1.36
NAGPA	1.4E-03	1.3E-03	3.2E-03	-1.08	2.21
NEU1	9.2E-02	4.1E-02	1.7E-01	-2.27	1.83
NEU2	4.1E-04	1.2E-04	7.9E-04	-3.37	1.94
NEU3	9.8E-03	6.6E-03	6.8E-02	-1.47	6.95
NEU4	2.5E-04	8.9E-05	2.4E-04	-2.83	-1.03
OGT	7.9E-02	4.7E-02	4.9E-02	-1.67	-1.6
POFUT1	3.6E-02	3.0E-02	2.0E-02	-1.19	-1.8
POFUT2	2.6E-02	1.8E-02	1.0E-02	-1.42	-2.64
POMGNT1	5.0E-02	3.9E-02	7.8E-02	-1.27	1.56
POMT1	8.2E-04	6.5E-04	2.7E-02	-1.25	32.84
POMT2	1.0E-02	2.2E-02	5.1E-02	2.16	5.16
PRKCSH	2.0E-01	1.8E-01	3.0E-01	-1.09	1.54
ST3GAL1	2.0E-01	9.9E-03	1.1E-02	-20.46	-17.78
ST3GAL2	4.7E-02	2.0E-02	4.3E-02	-2.35	-1.1
ST6GAL1	9.5E-03	1.9E-02	1.2E-03	2.02	-8.06
ST6GALNAC1	2.2E-03	1.6E-05	6.1E-05	-132.05	-35.81
ST8SIA2	2.2E-05	2.4E-05	8.8E-05	1.11	4.04
ST8SIA3	9.2E-05	1.2E-04	6.2E-05	1.27	-1.48
ST8SIA4	2.1E-05	6.2E-04	7.6E-03	28.94	353.97
ST8SIA6	2.1E-05	3.2E-05	2.3E-03	1.48	105.96
UGCGL1	5.3E-02	6.2E-02	1.2E-01	1.16	2.32
UGCGL2	3.1E-02	1.1E-02	2.6E-02	-2.79	-1.17

Supplemental Methods

Human tumor cell lines

HT29 human colorectal cancer cells and DU4475 human breast cancer cells were purchased from ECACC (Porton Down, UK). HOS osteosarcoma[1] as well as MeWo and MV3 melanoma[2] cells were kindly provided by the Dept. of Pediatric Hematology and Oncology (University Medical Center Hamburg-Eppendorf, UKE) and the Dept. of Dermatology at UKE, respectively. The human pancreatic cancer cell line PaCa5061[3] and gastric cancer cell line GC5023[4] were established in the Dept. of General, Visceral and Thoracic Surgery at UKE. The human small cell lung cancer cell lines SW2 and H69-AR were kind gifts from Prof. Zangemeister-Wittke (Institute of Pharmacology, University of Bern, Switzerland). The human multiple myeloma cell lines AMO-1 and IM-9 were kindly provided by Prof. M. Binder (Dept. of Oncology and Hematology, UKE). The human acute myeloid leukemia cell line Molm13 was provided by Dr. J. Wellbrock (Depot. of Oncology and Hematology, UKE). Human SKOV3 ovarian cancer cells were obtained from ATCC (Manassas, USA) and cultured in McCoy's 5A + L-glutamine supplemented with 10% FCS and 1% penicillin/ streptomycin (Gibco). All other human tumor cell lines were cultured in RPMI-1640 + L-glutamine (Gibco), supplemented with 10% FCS and 1% penicillin/ streptomycin and were kept under standard conditions (37°C, 95% H_2O-saturated atmosphere, 5% CO_2).

Human primary tumor models

The primary human colon cancer model PT457 was derived from a primary adenocarcinoma of the colon which was surgically resected from a male Caucasian in 2014. The PT1003 model was

established from a surgically resected, chemo-naïve liver metastasis of a colon sigmoideum adenocarcinoma from a 73 years-old male Caucasian. For spheroid culture, the protocol published by Jeppesen et al. was used.[5] Slight modifications of this protocol were the use of a 100 µm mesh size cell strainer for preparation and the use of Matrigel-coated 24-well plates for subsequent cultivation. Tumoroids were growing in 30-50µm domes surrounded by 1 mL stem cell medium. The use of patient material for research purposes was approved by the local ethics committee (Ärztekammer Hamburg, project PV4753, Sept 2nd, 2014).

Primary endothelial cells

Human umbilical vein endothelial cells (HUVEC) and human pulmonary microvascular endothelial cells (HPMEC) were obtained from PromoCell and cultured as described.[6,7] Murine pulmonary endothelial cells (MPEC) were freshly isolated as illustrated in Fig. 2D and previously described.[8] All experiments with primary cells were performed within the first six passages.

qRT-PCR for pulmonary Sele, Vcam1 and Icam1 expression

To analyze effects of the BZM treatment on pulmonary *Sele*, *Vcam1* and *Icam1* expression, xenograft tumor-bearing mice treated with PBS or BZM were sacrificed, lungs were resected and total RNA was extracted using a standard phenol/chloroform protocol and transcribed to cDNA using the cDNA RT^2 Easy First Strand Synthesis Kit (Qiagen). cDNA was used for qRT-PCR in a StepOnePLus system (Applied Biosystems, Thermo Fisher). *Gapdh* was used as housekeeping control. Nucleotide sequences of SYBR® Green primers were as follows:

Sele-fwd GGCTTTAGCTTGCATGGCTC, *Sele*-rev CATCTTTCCCGGGACGTCAA; *Icam1*-fwd CCATCCATCCCAGAGAAGCC, *Icam1*-rev CACTGAGTCTCCAAGCCCAG; *Vcam1*-*f*wd GTCACGGTCAAGTGTTTGGC, *Vcam1*-rev TGTTCATGAGCTGGTCACCC.

Ex vivo lung perfusion model

SCID mice were left untreated (control) or treated with 1 µg rmTNFα i.p. (Peprotech) for 4h. TNFα-treated mice were pretreated with PBS (solvent control) or 1.25 mg/kg BZM 24h and 1h before TNFα injection. 4h after TNFα injection, mice were narcotized and the cardiorespiratory system was resected and extracorporeally perfused as described[9] and illustrated in Fig. 2E. In short, 12 weeks old mice were narcotized and a cannula was placed into the trachea, by which the lungs were held under static inflation with a gas mixture of 30 % O_2, 5% CO_2 and 65 % N_2. Hence, normoxic conditions were maintained during the experiment, but ventilatory lung excursions were concomitantly avoided. Afterwards, the Vv. cavae were ligated, cannulae were inserted into the pulmonary artery and the left atrium, and the cardiorespiratory system was resected *en bloc*. The lung vasculature was then perfused through the cannulae with a HEPES-buffered perfusate at 37°C. Under these conditions, the lung periphery was examined using real-time epifluorescent video microscopy (Zeiss Axiotech with a Zeiss 40x water emission objective [NA: 0.8], Photometrics CoolSnap HQ camera). Physiological blood flow conditions in the lung microvasculature were controlled by adding isolated red blood cells (RBC) to the perfusate. Afterwards, $1x10^6$ HOS cells (labeled with Calcein RedOrange) were added to the perfusate and their adhesive behavior was analyzed in each compartment of the pulmonary microcirculation, namely precapillary arterioles, capillaries and post-capillary venules. RBC velocity and HOS

tumor cell adhesion or arrest was analyzed from the digital recordings using image analysis software (MetaMorph, Molecular Devices).

Single cell force spectroscopy

To measure the adhesion force [nN] between cancer and endothelial cells, we applied single cell force spectroscopy (SCFS). As described earlier,[10-12] this method is based on atomic force microscopy (AFM) and allows to quantify the adhesion force (nN) between single cancer cells and endothelial cells. The CellHesion® software (JPK, SPM, version 4) directed the experiment. Analysis of the data was performed using JPK Data Processing (software version 4.3.18). Briefly, 1.5 x 10^5 human umbilical vein endothelial cells (HUVECs) were seeded overnight in a well of an ibidi Culture-Insert (Culture-Insert 2 well, #80209, ibidi, Germany). The next day, the Culture-Inserts were removed and the cells were supplemented for 4 h with fresh medium, with or without IL-1α (10 ng/ml). Tipless cantilevers (#ARROW-TL1x48-10, NanoWorld AG) were preincubated for 20 min in PBS containing 1 mg/ml wheat germ agglutinin (WGA; #L-9640, Sigma-Aldrich). Before starting the experiment, HUVECs were rinsed 3x with Ringer's solution (in mmol/l: NaCl 122.5, KCl 5.4, $CaCl_2$ 1.2, $MgCl_2$ 0.8, HEPES 10, and D-glucose 5.5, adjusted to pH 7.4 with 1 M NaOH) and maintained in this solution. Afterwards, 2 µl of a cancer cell suspension (~100 cells/ µl) were added to a cell-free area within the dish containing the HUVECs. Next, the WGA-coated cantilever was guided under optical control over a single cancer cell and brought into contact with this cell for 6 s by pressing the cantilever onto the cell with a maximal loading force of 2 nN. To ensure that the cancer cells were attached to the cantilever and always had the same mechanical properties, we standardized this picking procedure.

For measuring the cell-cell adhesion forces, the parameters were changed to 2 s contact time with a maximum loading force of 1 nN. This time, the cantilever with the attached cancer cell was lowered under optical control onto a single HUVEC within the endothelial cell layer and after 2 s contact time the cantilever was lifted back to its starting position. The maximal adhesion force was determined from the resulting force-distance curve. Each cancer cell was brought into contact with 20 individual HUVECs (n=1). At least three independent experiments were performed for each condition (n≥3). Further parameters for the measurements were as follows: pulling length (z-length) was set to 100 µm (to ensure a complete separation of cells), velocity during approach and retraction was set to 5 µm/s. The spring constant was 0.03 N/m and had been corrected by the Standardized Nanomechanical AFM Procedure (SNAP).[13]

shRNA-mediated depletion of CD44, E-selectin, C2GNT2 and MGAT5

To test the functional importance of CD44 and MGAT5 (HOS and MeWo cells), E-selectin (HUVECs) and C2GNT2 (HT29 and PaCa5061 cells) the mRNAs of interest were stably depleted by lentiviral transduction of corresponding shRNAs. In case of the CD44 knockdown, a pLVX-puro vector was used and continuous selection with puromycin, FACS and limiting dilution cloning were performed. Finally, at least 5 clones showing the strongest depletion were pooled to avoid clonal effects. In case of the E-selectin and C2GNT2 knockdowns, cells were transduced with a pLVX-puro vector containing the shRNA of interest and subsequently selected with puromycin, but without FACS or limiting dilution cloning. In case of MGAT5, knockdown cells were transduced and selected in the same way, but additionally FACS-sorted based on cell surface PHA-L binding. In parallel, the 'shControl' cell lines were generated by transducing parental cells with a pLVX-puro vector containing a nonsense sequence (without mRNA target in mammalian

cells). The nucleotide sequences of the used shRNAs were: shCD44: 5'-GGCGCAGATCGATTTGAAT-3'; shE-selectin: 5'-CACACACCTGGTTGCAATT-3'; shC2GNT2 (GCNT3): 5'-CCGGGCTTAGAA-GAATACCTACGTTCTCGAGAACGTAGGTATTCTTCTAAGCTTTTTG-3'; shMGAT5: 5'-GATCCGGCGGAAATTCGTACAGATTTCAAGAGAATCTGTACGAATTTCCGCCTTTTT TACGCGTG-3'.

CAM and glycosyltransferase expression in human tumor cell lines

Commonly described E-selectin ligand carrier glycoproteins were tested on HT29, PaCa5061, HOS, and MeWo cells by flow cytometry using commercial antibodies against CD44 (B-F24), CD44v3 (3G5), CD24 (eBioSN3), CD43 (eBio84-3C1), ESL-1 (AE-6), MUC-1 (SantaCruz), PSGL-1 (FLEG), LGALS3BP (3G8). Isotype controls were used as appropriate. Tumor cells were marked dead or alive by propidium iodide staining immediately before flow cytometry.

RNA was extracted from HT29, HOS and MeWo cells and human glycosyltransferase qPCR profiler arrays performed as described.[14] In short, RNA concentrations were quantified using a NanoDrop spectrophotometer and 1 mg was processed to cDNA with RT2 First Strand Kit (SA Biosciences). Expression levels of 84 human glycosylation genes were determined with the Human Glycosylation RT^2 Profiler PCR Array (SA Biosciences) in a LightCycler 480 (Roche). Housekeeping genes for normalization and internal controls for genomic DNA contamination, RNA quality, and general PCR performance were included as well. Differences in C2GNT2 (GCNT3) expression as indicated by the qPCR profiler array were validated by WB as described.[15] Total protein extraction was made with RIPA lysis buffer. For protein detection, the following

antibodies were used: polyclonal rabbit anti-GCNT3 (C2GNT2), mouse monoclonal anti-HSC70 (B-6) (SantaCruz).

Lectin flow cytometry

α-2,3- and α-2,6-sialic acid residues within the human tumor cell glycocalyx were determined using biotinylated *Maackia amurensis* (MAA-II) and *Sambucus nigra* (SNA-I) lectin (Vector Labs.) at 10 µg/mL in lectin buffer (Tris-buffered saline +Ca^{2+}/ +Mg^{2+}) for 30 min at 4°C. As 'isotype' controls, lectins were applied after treating the tumor cells with periodic acid. Lectins were labeled with streptavidin-APC (Sigma) for flow cytometry. Tumor cells were treated with neuraminidase (see methods of main text) or left untreated prior to the lectin binding assay.

CD44 WB, enzymatic treatments and lectin pulldown assays

HOS and MeWo cell protein extracts from parental cell lines as well as MGAT5 control and knockdown derivatives were generated by scraping the cells in RIPA buffer (supplemented with protease inhibitor cocktail (Calbiochem) and x M vanadate, 30 min at 4°C, then 30 min centrifuged at 14,000 g at 4°C). Protein extracts were left untreated or treated with PNGase F (*Flavobacterium meningosepticum*, recombinant from *E. coli*; 1 U/ 10 µg protein overnight at 37°C) or sialidase (*Arthrobacter ureafaciens*, 1.25 mU/ 10 µg protein for 45 min at 37°C) before protein bands were separated using SDS-PAGE followed by anti-CD44 western blot analysis. Briefly, proteins were transferred to PVDF membranes and unspecific binding sites were blocked with blocking solution (StartingBlock™ Blocking Buffer) + 0.05% Tween 20 for 1h at RT. Primary antibody (R&D systems, clone 2C5, 1 µg/mL) was incubated for 1 h at RT. HRP-conjugated goat-anti-mouse was

used as secondary antibody and binding was detected using a chemiluminescence kit (Thermo Fisher). Additional tumor cell samples were treated with 2 µM synthetic swainsonine for 72 h prior to protein extraction (control: methanol). In addition, some protein extracts (+/- swainsonine or from MGAT5 control and knockdown derivatives) were incubated with DSL-agarose or PHA-L agarose (both from Vector Labs.) in a protein: lectin ratio of 10: 1 overnight at 4°C (rolling). After centrifugation and washing five times, the precipitated proteins were subjected to SDS-PAGE and CD44 WB as described above.

Immunohistochemistry

Immunohistochemistry for hNCAM was carried out on 4 µm lung tissue sections (FFPE) from SW2 xenograft mice. After de-paraffinization, antigen retrieval was performed with Dako S1699 Retrieval Solution at pH 6 in a microwave for 2 x 4 min. Final concentration of the primary antibody (Leica #NCL-CD56-1B6) was 0.88 µg/ml and visualization of bound antibody was performed with the Dako Real Detection System K5005.

5 µm sections of HT29, PaCa5061, HOS and MeWo xenograft tumors (FFPE) were stained with anti-Ki67 (Dako S1699 Retrieval Solution at pH 6, 85°C overnight; primary antibody: Dako #M7240, final concentration: 1.1 µg/ml; visualization with the Vectastain ABC-AP kit from Vector Labs.), anti-pH2AX (Dako S1699 Retrieval Solution at pH 6, 121°C, 10 min; primary antibody: abcam #ab81299, final concentration: 0.07 µg/ml; visualization with ABC-AP), and anti-CD31 (Dako S2367 Retrieval Solution at pH 9, 121°C, 10 min; primary antibody: abcam #ab28364, final concentration: 0.8 µg/ml, visualization with ABC-AP). For quantification of the Ki67 and pH2AX staining, the stained area was determined using Image J software (Color

Threshold Plugin) analyzing up to 27 viewing fields (200-fold magnification) depending on the entire size of the respective xenograft tumor section. Three samples per group (PBS vs. BZM) were considered per xenograft tumor entity (HT29, PaCa5061, HOS, MeWo). For quantification of the CD31 staining, the number of microvessels (CD31-positive) was counted in up to 20 viewing fields (200-fold magnification) per tumor depending on the tumor size and three samples were considered per group.

Immunohistochemistry for sLeA was performed on 5 µm sections of agar-embedded SKOV3 cells as well as SKOV3, HOS and MeWo xenograft tumors (FFPE). In addition, sLeA was stained on colon cancer and ovarian cancer surgical specimens. In short, sections were de-paraffinized and pre-treated with trypsin 0.1% in a water bath at 37°C for 10 min. The primary antibody was 121SLE from abcam (#ab3982) diluted 1:750 and bound antibody was visualized using the Dako Real Detection System K5005. Counterstaining of nuclei was performed with Mayer's hemalum solution for 3-5 s.

Patient material

Clinical colorectal cancer and ovarian cancer specimens were collected from surgically removed tissues adhering to guidelines of the local ethical review board and after written informed consent of the patients (ethics committee of the Universität zu Lübeck, approval #07-124). For sLeA immunohistochemistry, tissues of 60 colorectal cancers as well as 30 corresponding adjacent normal mucosa specimens from the same patients were selected. Tissue cores (diameter 1.5 mm) were implemented into a tissue microarray by using a semiautomated arrayer (TMArrayer, Pathology Devices, San Diego, USA) as described [16]. The ovarian cancer specimens were provided

by Professor Johannes Dietl (Department of Obstetrics and Gynecology, Würzburg University Hospital).

Statistics

Data are presented as means ± SD unless otherwise indicated. Student's t and Mann-Whitney-U tests were performed as appropriate to assess associations between variables. Statistical significance was assigned at 2-tailed p-values less than 0.05. Numbers of lung metastases were also evaluated by an analysis of covariance (ANCOVA) with the tumor weight (Fig. 6A) or growth period (Fig. 2B) as covariates. These statistical tests were carried out using IBM SPSS software (SPSS version 21.0 for Windows, IBM). All data were visualized using Graph Pad Prism 5.0 (Graph Pad Software, San Diego).

Supplemental References

1. McAllister, R.M., Gardner, M.B., Greene, A.E., Bradt, C., Nichols, W.W., and Landing, B.H. (1971). Cultivation in vitro of cells derived from a human osteosarcoma. Cancer *27*, 397-402.
2. Carey, T.E., Takahashi, T., Resnick, L.A., Oettgen, H.F., and Old, L.J. (1976). Cell surface antigens of human malignant melanoma: mixed hemadsorption assays for humoral immunity to cultured autologous melanoma cells. Proc Natl Acad Sci U S A *73*, 3278-3282.
3. Kalinina, T., Gungor, C., Thieltges, S., Moller-Krull, M., Penas, E.M., Wicklein, D., et al. (2010). Establishment and characterization of a new human pancreatic adenocarcinoma cell line with high metastatic potential to the lung. BMC Cancer *10*, 295. 1471-2407-10-295.
4. Starzonek, S., Maar, H., Labitzky, V., Wicklein, D., Rossdam, C., Buettner, F.F.R., et al. (2020). Systematic analysis of the human tumor cell binding to human vs. murine E- and P-selectin under static vs. dynamic conditions. Glycobiology. 10.1093/glycob/cwaa019.
5. Jeppesen, M., Hagel, G., Glenthoj, A., Vainer, B., Ibsen, P., Harling, H., et al. (2017). Short-term spheroid culture of primary colorectal cancer cells as an in vitro model for personalizing cancer medicine. PLoS One *12*, e0183074. 10.1371/journal.pone.0183074.
6. Richter, U., Schroder, C., Wicklein, D., Lange, T., Geleff, S., Dippel, V., et al. (2011). Adhesion of small cell lung cancer cells to E- and P-selectin under physiological flow conditions: implications for metastasis formation. Histochem Cell Biol *135*, 499-512. 10.1007/s00418-011-0804-4.
7. Lange, T., Kupfernagel, M., Wicklein, D., Gebauer, F., Maar, H., Brugge, K., et al. (2014). Aberrant presentation of HPA-reactive carbohydrates implies Selectin-independent metastasis formation in human prostate cancer. Clin Cancer Res *20*, 1791-1802. 10.1158/1078-0432.CCR-13-2308.
8. Fehrenbach, M.L., Cao, G., Williams, J.T., Finklestein, J.M., and Delisser, H.M. (2009). Isolation of murine lung endothelial cells. Am J Physiol Lung Cell Mol Physiol *296*, L1096-1103. 10.1152/ajplung.90613.2008.
9. Kiefmann, R., Rifkind, J.M., Nagababu, E., and Bhattacharya, J. (2008). Red blood cells induce hypoxic lung inflammation. Blood *111*, 5205-5214. 10.1182/blood-2007-09-113902.
10. Bulk, E., Kramko, N., Liashkovich, I., Glaser, F., Schillers, H., Schnittler, H.J., et al. (2017). KCa3.1 channel inhibition leads to an ICAM-1 dependent increase of cell-cell adhesion between A549 lung cancer and HMEC-1 endothelial cells. Oncotarget *8*, 112268-112282. 10.18632/oncotarget.22735.
11. Hofschroer, V., Koch, K.A., Ludwig, F.T., Friedl, P., Oberleithner, H., Stock, C., et al. (2017). Extracellular protonation modulates cell-cell interaction mechanics and tissue invasion in human melanoma cells. Sci Rep *7*, 42369. 10.1038/srep42369.
12. Lindemann, O., Rossaint, J., Najder, K., Schimmelpfennig, S., Hofschroer, V., Walte, M., et al. (2020). Intravascular adhesion and recruitment of neutrophils in response to CXCL1 depends on their TRPC6 channels. J Mol Med (Berl) *98*, 349-360. 10.1007/s00109-020-01872-4.
13. Schillers, H., Rianna, C., Schape, J., Luque, T., Doschke, H., Walte, M., et al. (2017). Standardized Nanomechanical Atomic Force Microscopy Procedure (SNAP) for Measuring Soft and Biological Samples. Sci Rep *7*, 5117. 10.1038/s41598-017-05383-0.
14. Lange, T., Ullrich, S., Muller, I., Nentwich, M.F., Stubke, K., Feldhaus, S., et al. (2012). Human prostate cancer in a clinically relevant xenograft mouse model: identification of beta(1,6)-branched oligosaccharides as a marker of tumor progression. Clin Cancer Res *18*, 1364-1373. 1078-0432.CCR-11-2900.
15. Oliveira-Ferrer, L., Kurschner, M., Labitzky, V., Wicklein, D., Muller, V., Luers, G., et al. (2015). Prognostic impact of transcription factor Fra-1 in ER-positive breast cancer: contribution to a metastatic phenotype through modulation of tumor cell adhesive properties. J Cancer Res Clin Oncol. 10.1007/s00432-015-1925-2.
16. Gemoll, T., Roblick, U.J., Szymczak, S., Braunschweig, T., Becker, S., Igl, B.W., et al. (2011). HDAC2 and TXNL1 distinguish aneuploid from diploid colorectal cancers. Cell Mol Life Sci *68*, 3261-3274. 10.1007/s00018-011-0628-3.
17. Schweitzer, K.M., Drager, A.M., van der Valk, P., Thijsen, S.F., Zevenbergen, A., Theijsmeijer, A.P., et al. (1996). Constitutive expression of E-selectin and vascular cell adhesion molecule-1 on endothelial cells of hematopoietic tissues. Am J Pathol *148*, 165-175.
